# Creeping fat exhibits distinct Inflammation-specific adipogenic preadipocytes in Crohn’s disease

**DOI:** 10.3389/fimmu.2023.1198905

**Published:** 2023-12-04

**Authors:** Nahee Hwang, Dongwoo Kang, Su-Jin Shin, Bo Kyung Yoon, Jaeyoung Chun, Jae-woo Kim, Sungsoon Fang

**Affiliations:** ^1^ Department of Biochemistry and Molecular Biology, Yonsei University College of Medicine, Seoul, Republic of Korea; ^2^ Graduate School of Medical Science, Brain Korea 21 Project, Yonsei University College of Medicine, Seoul, Republic of Korea; ^3^ Chronic Intractable Disease for Systems Medicine Research Center, Yonsei University College of Medicine, Seoul, Republic of Korea; ^4^ Department of Medicine, Yonsei University College of Medicine, Seoul, Republic of Korea; ^5^ Department of Pathology, Gangnam Severance Hospital, Yonsei University College of Medicine, Seoul, Republic of Korea; ^6^ Department of Internal Medicine, Gangnam Severance Hospital, Yonsei University College of Medicine, Seoul, Republic of Korea; ^7^ Department of Biomedical Sciences, Gangnam Severance Hospital, Yonsei University College of Medicine, Seoul, Republic of Korea

**Keywords:** creeping fat, Crohn’s disease, inflammatory bowel disease, fat fibrosis, inflammation, pentraxin-3, preadipocytes, fibroblast

## Abstract

Creeping fat (CrF) is an extraintestinal manifestation observed in patients with Crohn’s disease (CD). It is characterized by the accumulation of mesenteric adipose tissue (MAT) that wraps around the intestinal wall. Although the role of CrF in CD is still debated, multiple studies have highlighted a correlation between CrF and inflammation, as well as fibrostenosais of the intestine, which contributes to the worsening of CD symptoms. However, the mechanism underlying the potential role of CrF in the development of Crohn’s fibrosis remains an enigma. This study aimed to analyze CrF comprehensively using single-cell RNA sequencing analysis. The data was compared with transcriptomic data from adipose tissue in other disease conditions, such as ulcerative colitis, lymphedema, and obesity. Our analysis classified two lineages of preadipocyte (PAC) clusters responsible for adipogenesis and fibrosis in CrF. Committed PACs in CrF showed increased cytokine expression in response to bacterial stimuli, potentially worsening inflammation in patients with CD. We also observed an increase in fibrotic activity in PAC clusters in CrF. Co-analyzing the data from patients with lymphedema, we found that pro-fibrotic PACs featured upregulated pentraxin-3 expression, suggesting a potential target for the treatment of fibrosis in CrF. Furthermore, PACs in CrF exhibited a distinct increase in cell-to-cell communication via cytokines related to inflammation and fibrosis, such as CCL, LIGHT, PDGF, MIF, and SEMA3. Interestingly, these interactions also increased in PACs of the lymphedema, whereas the increased MIF signal of PACs was found to be a distinct characteristic of CrF. In immune cell clusters in CrF, we observed high immune activity of pro-inflammatory macrophages, antigen-presenting macrophages, B cells, and IgG^+^ plasma cells. Finally, we have demonstrated elevated IgG^+^ plasma cell infiltration and increased pentraxin-3 protein levels in the fibrotic regions of CrF in CD patients when compared to MAT from both UC patients and healthy individuals. These findings provide new insights into the transcriptomic features related to the inflammation of cells in CrF and suggest potential targets for attenuating fibrosis in CD.

## 1 Introduction

Crohn’s disease (CD) and ulcerative colitis (UC) are two major subtypes of inflammatory bowel disease: chronic and relapsing conditions that affect a significant number of individuals worldwide (1, 2). Although CD and UC share some clinical features, they differ in several aspects, including the distribution and extent of inflammation within the gastrointestinal tract (3, 4). One notable distinction between the two is the occurrence of “creeping fat” (CrF) in patients with CD. CrF is a type of adipose tissue that infiltrates and envelops inflamed intestinal segments (5). Recent studies have found a link between the characteristics of CrF and the development of CD (6–8). Some studies suggest that CrF can protect against bacterial translocation and intestinal inflammation, while others suggest that it can contribute to the progression of the disease by triggering an uncontrolled inflammatory response. Additionally, the buildup of CrF has been identified as a risk factor for post-surgical recurrence in CD, leading to the development of new surgical techniques such as mesentery exclusion (9–13). However, the mechanisms behind the formation of CrF and its connection to inflammation and fibrostenosis of CD are not yet fully understood.

Adipose tissue harbors diverse cell types, including adipocytes, preadipocytes, endothelial cells, smooth muscle cells, stromal cells, and immune cells (14). These cells play a crucial role in regulating inflammation by producing cytokines and interacting with each other (15). In CrF, pro-inflammatory and pro-fibrotic cytokines, such as TNF-α and IL-6, and adipokines, such as resistin and leptin, are highly enriched compared to normal adipose tissue (16–18). Recently, studies using single-cell RNA sequencing have emerged to explore the transcriptomic features of constituent cells in CrF (19, 20). They identified an increase in bacteria recognizing M1 macrophages and pro-fibrotic M2 macrophages in CrF. Additionally, specific subclusters of cells, including vascular endothelial cells (VECs), fibroblasts, and myeloid cells, have been found to play a role in the inflammation and fibrosis of CrF. VECs with high expression of lipoprotein lipase show an increased expression of genes related to bacterial responses, indicating their involvement in the immune response against bacteria. Inflammatory fibroblasts producing IL-1β and NK-κB have been found to contribute to collagen synthesis and the accumulation of extracellular matrix, leading to fibrosis in CrF. Preadipocytes are known to contribute to fibrosis through the up-regulation of genes related to extracellular matrix (ECM) accumulation in CrF (19). Their roles in fibrosis and inflammation have been observed in other diseases, such as type 2 diabetes, liver fibrosis, and systemic sclerosis (21–23). However, the molecular characteristics of preadipocytes in CrF are still unclear.

Despite the emerging research on the characteristics of CrF, there is still a need to better understand the transcriptomic features of its constituent cells, particularly at the single-cell level. In this study, we investigated the transcriptomic characteristics of each cell type present in CrF of CD patients, with a specific focus on preadipocytes at various stages of differentiation. Our study aims to uncover these mechanisms and identify potential therapies.

## 2 Materials and methods

### 2.1 Quality control, data integration, and unsupervised clustering

**Figure 1:**
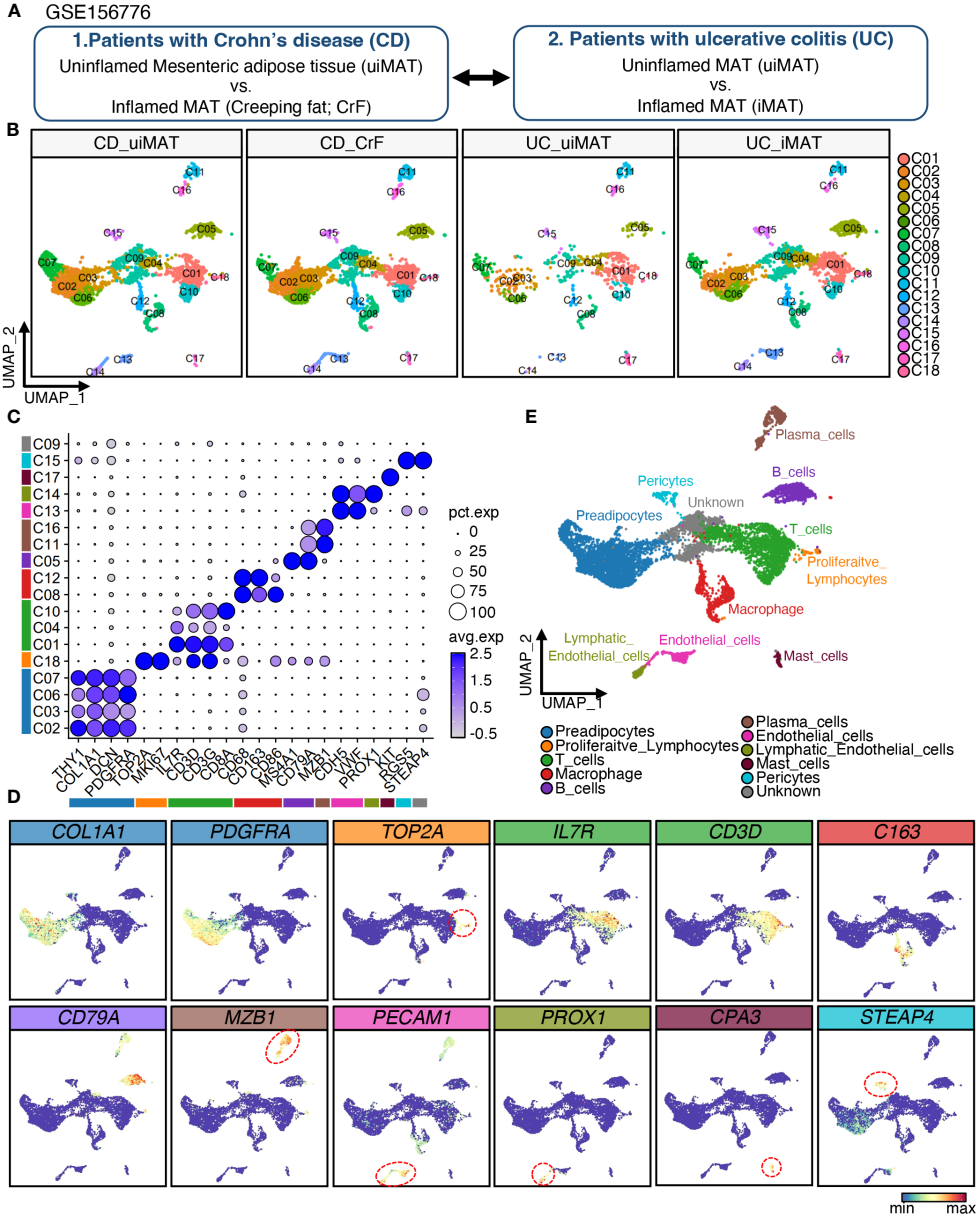
Single-Cell RNA-Seq Reveals Cellular Diversity and Heterogeneity in Mesenteric Adipose Tissue of Patients with CD and UC. **(A)** Schematic representation of the experimental procedure: Uninflamed mesenteric adipose tissue (n = 3; CD_uiMAT) and inflamed mesenteric adipose tissue (n = 3; CD_CrF) from patients with Crohn’s disease, as well as uninflamed mesenteric adipose tissue (n = 2; UC_uiMAT) and inflamed mesenteric adipose tissue (n = 2; UC_iMAT) from patients with ulcerative colitis, were obtained from GSE156776 **(B)** A uniform manifold approximation projection (UMAP) plot revealed 18 clusters of 8378 cells. **(C, D)** Dot plots and feature plots were used to visualize the expression of established marker genes for each lineage in each cluster. **(E)** UMAP plot showing the annotation derived from panels **(C, D)**.

The single-cell RNA sequencing (scRNA-seq) dataset of CrF was accessed from the public GEO database under accession number GSE156776. The dataset was comprised of paired MAT attached to inflamed or uninflamed ileum from three CD patients and two UC patients undergoing bowel resection (19). The R package Seurat (4.2.1) was used for quality control, clustering, and data integration (24). The same inclusion criteria for cells with more than 200 features, 200 counts, and less than 5% mitochondrial genes were applied to all the datasets in this study. The filtered data were log-normalized with a scale factor of 10,000, using the NormalizeData function. Normalized individual datasets from patients with CD and UC were combined using the R package Seurat. The SelectIntegrationFeatures function from the R package Seurat was used to select features repeatedly variable across datasets. Subsequently, the FindIntegrationAnchors function was used to determine the integration anchors for the integration of datasets. Individual datasets were merged into a single Seurat object using the IntegrateData function with predetermined integration anchors. The RunHarmony function in the R package was used to eliminate batch effects from different samples. To select the top 2000 highly variable features, we applied the FindVariableFeature function with a variance-stabilizing transformation to the integrated Seurat object. Prior to the dimensional reduction, the data were scaled using the ScaleData function. The RunPCA function was applied to the scaled data using previously identified highly variable features to determine the principal components (PCs). The selected top 50 PCs were used for further clustering analyses. The data were clustered based on the shared nearest-neighbor graph generated using the FindNeighbors function. After determining the nearest neighbors, the cells were clustered by the Louvain algorithm using the FindClusters function with the resolution parameter set to 1. To graph the uniform manifold approximation and projection (UMAP) plot, the RunUMAP function was used with previously defined PCs. The integrated dataset consisted of 8373 cells that were clustered into 20 clusters. Each cluster was annotated by identifying cluster markers using the FindAllMarkers function in the Seurat package. The cluster markers were matched with reference markers for each cell type, as shown in **Figure 1C**. After annotation of each cell cluster, immune cells were subgrouped by lineage for subclustering. The subclustering process was identical to that of the UMAP clustering process, with 20 as the dimension parameter of reduction and 0.7 as the resolution parameter. Preadipocyte clusters were subclustered following the same procedure as the immune cells.

### 2.2 Differential gene expression analysis

To identify differentially expressed genes (DEGs) for each subcluster, the Seurat FindAllMarkers function based on the Wilcoxon rank-sum test was applied to the integrated data. Genes with a Bonferroni-corrected p-value < 0.05 and an absolute value of log_2_ of the fold change > 0.2 were considered significant. The significant DEGs for each cluster were input into the enrichGO function of the clusterProfiler (4.6.0) package to assess the activated pathways in each cluster (25). Significantly upregulated pathways were defined as gene ontology of biological process with Benjamini–Hochberg adjusted p-values < 0.05. The results of the DEG analysis were plotted as a bar plot and enrichment map using the emapplot function.

### 2.3 Preadipocyte cluster comparison

The clustered PAC clusters were compared to scRNA-seq dataset on adipose tissues obtained from five patients with secondary lymphedema in thigh after cervical cancer surgery. The annotated scRNA-seq dataset of the cancer-associated lymphedema dataset was provided by the author of the original article (26). The annotated single-nucleus RNA sequencing data of visceral adipose tissue from obese individual was downloaded from Gene Expression Omnibus (GSE176171) (27). The spatial transcriptomic dataset on periumbilical subcutaneous white adipose tissues from lean and obese individuals were obtained from Mendeley Data and analyzed using Seurat (28). The PAC clusters were compared to those from individuals with lymphedema and spatial transcriptomic dataset by calculating the similarity of clusters from different origins. The top 10 upregulated DEGs for each cluster in the lymphedema data were selected as module genes for each cluster, and the top 15 upregulated DEGs for each cluster in the obese spatial transcriptomics dataset were selected as module genes. To calculate the similarity of the subclusters, the Seurat AddModuleScore function was used to calculate the module score for each subtype of PACs based on the lymphedema and obese spatial transcriptomic PAC clusters.

### 2.4 RNA velocity analysis

The RNA velocity was determined by analyzing the relative abundance of spliced and unspliced RNA, thereby estimating the direction and rate of cellular differentiation. Raw FASTQ files for the identical dataset available from the Sequence Read Archive under the project accession number SRP278645 were used for RNA velocity analysis. Paired FASTQ files for each sample were used as inputs for ddSeeker to generate bam files tagged with cell barcodes and unique molecular identifiers (UMI) (29). The samtools (1.16.1) bam2fq function was used to generate single-ended FASTQ files from the unmapped bam files, and the single-ended FASTQ was aligned to the reference genome GRCh38.p13 using STAR (2.7.10b) (30–32). The aligned bam file was then merged with the unmapped bam files using the MergeBamAlignment function of the Picard package (2.27.5) (33). The merged bam file was input to the velocyto run function of the Python module velocyto (0.17), and the generated loom files were used as input for the scvelo (0.2.5) pipeline (34, 35). After separately generating loom files for each FASTQ file, the loom data were imported and merged into a single annotated loom file. The integrated Seurat data was imported into the annotated data format and merged with the annotated loom. Cellular dynamics data were recovered from the merged data using the tl.recover_dynamics function, and the RNA velocity for each cell was computed in the dynamical mode of the tl.velocity function in the scvelo package. The estimated RNA velocity was mapped onto a UMAP plot with subtype annotations. All analyses for mapping RNA velocity were performed in Linux 20.04 or python 3.8.8.

### 2.5 Intercellular interaction analysis

Intercellular interactions between cell clusters were analyzed using the R package CellChat (1.6.0) by computing the expression patterns of ligand-receptor pairs (36). The computeCommunProb function was applied to the clustered data using the trimean as the average expression level for each cluster to infer the interaction strength. The netAnalysis_signalingRole_scatter function was used to generate a scatter plot of the incoming and outgoing signaling interaction strengths. The rankNet function was applied to the dataset to compare the strength of information flow between inflamed and uninflamed tissues. The Wilcoxon test was used to identify significantly enriched signaling ligand-receptor pairs with p-value < 0.05. Signaling pathways that were upregulated only in the CrF data were plotted using the netVisual_individual function to visualize cell-to-cell interactions between cell clusters.

### 2.6 Statistical analysis

DEGs for each subcluster was defined as genes with two-sided Bonferroni adjusted p-value < 0.05, and upregulated pathways were defined using Benjamini-Hochberg adjusted p-values < 0.05. Intercellular interaction was analyzed using the Wilcoxon test, and significantly enriched ligand-receptor pair was defined as pairs with p-value < 0.05. All tests were subjected to the same criteria for statistical significance, with p < 0.05 being regarded as statistically significant. We denoted significance levels as follows: * (p < 0.05), ** (p < 0.01), and *** (p < 0.001).

### 2.7 Ethical and legal considerations

The study protocol was approved by the institutional review board at Gangnam Severance Hospital, Yonsei University of Korea (approval number: 3-2023-0331). The study complies with the Declaration of Helsinki and the principles of Good Clinical Practice.

### 2.8 Histological and immunohistochemistry analysis

We randomly selected 3 patients with Crohn’s disease, 3 patients with ulcerative colitis, and 1 patient with diverticulitis (with normal colonic mucosa) who underwent surgical resection at Gangnam Severance Hospital between January 2022 and January 2023. We reviewed all hematoxylin and eosin (H&E) slides used at the time of diagnosis. We selected regions with creeping fat in Crohn’s disease, mucosal ulceration in ulcerative colitis, and normal colonic mucosa in diverticulitis for Masson’s trichrome and immunohistochemistry by light microscopy. Masson’s trichrome and IHC staining were performed on 4 μm sections obtained from selected formalin-fixed paraffin-embedded (FFPE) blocks. The IHC staining for PTX3 (1:1000, sc-373951, mouse monoclonal, Santa Cruz Biotechnology, Santa Cruz, CA, USA), and CD138 (1:1000, EPR6454, rabbit monoclonal, Abcam, Cambridge, UK) was performed using Benchmark^®^ automatic immunostaining device (Roche Tissue Diagnostics, Tucson, USA) and an UltraViewTM Universal DAB Detection Kit (Ventana Medical Systems, Tucson, USA), according to the manufacturer’s instructions.

## 3 Results

### 3.1 Single-cell RNA-seq reveals cellular diversity and heterogeneity in mesenteric adipose tissue of patients with CD and UC

To investigate the cellular diversity and gene expression profiles of CrF in patients with CD, we analyzed scRNA-seq data of mesenteric adipose tissue (MAT) adjacent to both inflamed and uninflamed regions in three patients with CD and two patients with UC (**Figure 1A**). We will refer to the mesenteric adipose tissue (MAT) adjacent to inflamed and uninflamed intestine in UC patients as iMAT and uiMAT, and to the MAT adjacent to inflamed and uninflamed intestine in CD patients as CrF and uiMAT, respectively. The data were deposited in the GEO database under the accession number GSE156776. After filtering out low-quality cells, 8373 cells were included in further analysis. Unbiased UMAP clustering resulted in 18 clusters (**Figure 1B**), and each cluster was annotated using cell type-specific markers into 10 different lineages of PACs, proliferative lymphocytes, T cells, macrophages, B cells, plasma cells, endothelial cells, lymphatic endothelial cells, mast cells, pericytes, and a single cluster of unidentified cell types (**Figures 1C–E**). Four clusters (C02, C03, C06, and C07) expressed *PDGFRA, THY1, COL1A1*, and *DCN*, which are markers of PACs, and one cluster (C18) expressed *TOP2A* and *MKI67*, which are marker genes for proliferative lymphocytes. Three clusters (C01, C04, and C10) of T cell lineages expressed *IL7R, CD3D, CD3G*, and *CD8A*, which are markers for T cells, and two clusters (C08, C12) of macrophages expressed *CD68, CD163*, and *CD86*. A cluster (C05) of B cells expressed *MS4A1* and *CD79A*, and two clusters (C11 and C16) of plasma cells expressed *MZB1* as a marker gene. A cluster (C13) of endothelial cells expressed *CDH5* and *VWF*, whereas a cluster (C14) of lymphatic endothelial cells expressed *PROX1*. A cluster (C17) expressed mast cell markers, such as *KIT*, and a cluster (C15) of pericytes expressed *RGS5* and *STEAP4* (**Figures 1B, C**).

### 3.2 Analysis of differentially expressed genes reveals distinct transcriptomic characteristics of immune cell subclusters in CrF

**Figure 2:**
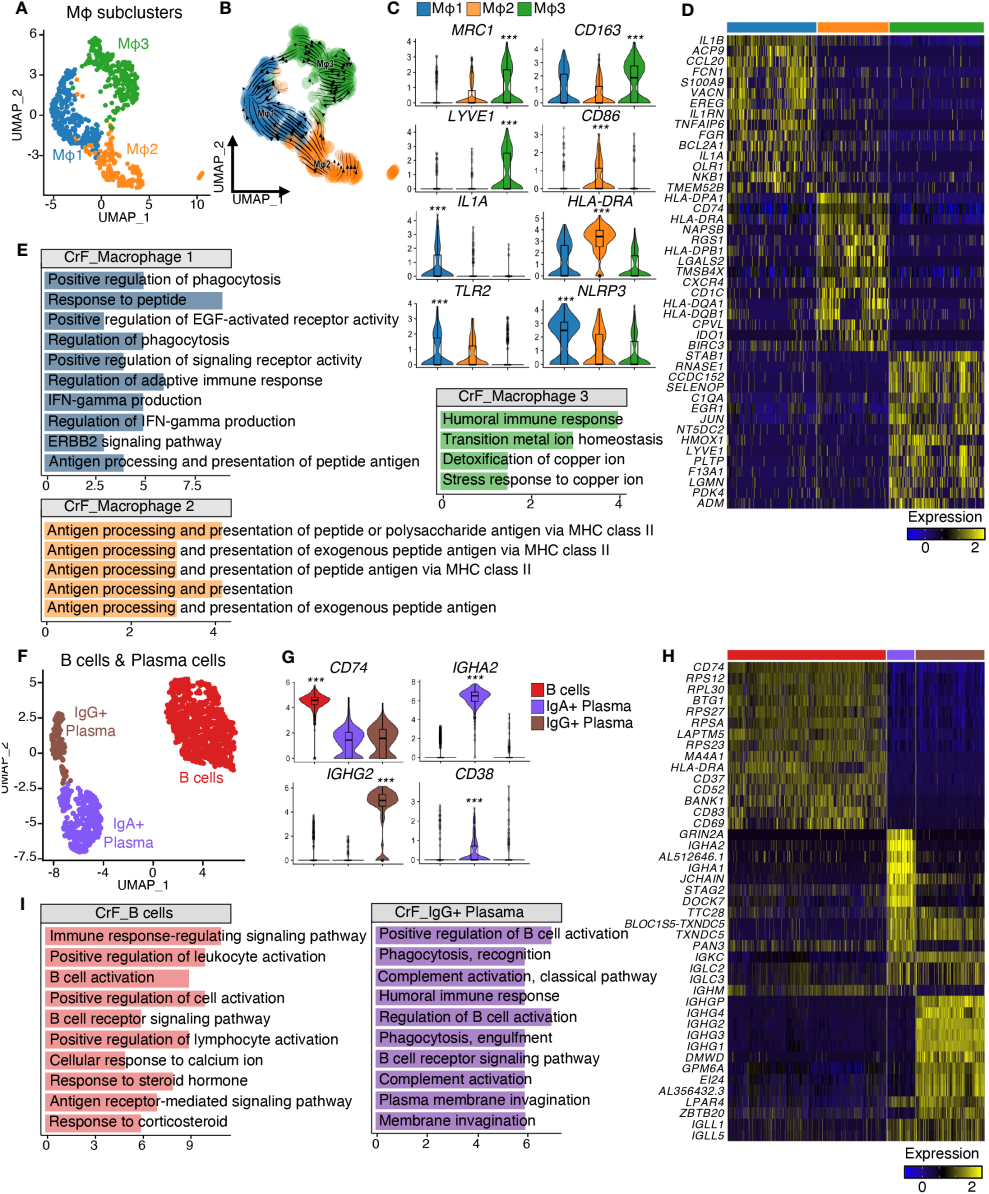
Analysis of Differentially Expressed Genes Reveals Distinct Transcriptomic Characteristics of Immune Cell Subclusters in CrF. **(A)** UMAP plot shows the macrophages isolated from **Figure 1E**, and the cluster analysis revealed three distinct clusters. **(B)** RNA-velocity analysis was performed on the macrophage clusters, with the velocity field projected onto the UMAP plot from **(A)**. The arrows depict the local average velocity assessed on a regular grid, indicating the extrapolated future states of cells. **(C)** Violin plots showing the RNA expression levels of selected cluster markers for specific cell clusters. **(D)** Distinct expression profiles of the three subpopulations of macrophages **(E)** Enriched Gene Ontology terms of the molecular signature for each subpopulation, hypergeometric test, adjusted p < 0.01. **(F)** UMAP plot shows the B cells and plasma cells isolated from **Figure 1E**, and the cluster analysis revealed three distinct clusters. **(G)** Violin plots showing the RNA expression levels of selected cluster markers for specific cell clusters. **(H)** Distinct expression profiles of the three subpopulations of B cells and plasma cells **(I)** Enriched Gene Ontology terms of the molecular signature for each subpopulation, hypergeometric test, adjusted p < 0.01. *** adjusted p < 0.001.

After annotating each cluster as a specific cell type, we performed subclustering on immune cells grouped by the lineage of differentiation. The subclustering of macrophage clusters resulted in three subpopulations, Mφ1, Mφ2, and Mφ3 (**Figure 2A**), each exhibiting distinct molecular signatures (**Figures 2C, D**). The Mφ1 population was confirmed to be the most polarized (**Figure 2B**) and displayed an increase in pro-inflammatory cytokines, including *IL1A, IL1B*, and *NLRP3*, suggesting that it is a pro-inflammatory macrophage population (37, 38). The Mφ2 population expressed marker genes for both M1- and M2-polarized macrophages, including *CD86* and *MRC1*, respectively (39), and had a high expression of antigen-presenting genes, including *CD1C* and MHC class II genes (40), designated as antigen-presenting macrophages. In contrast, Mφ3 expressed high levels of M2-polarized macrophage markers, such as *MRC1* and *CD163* (41), and displayed high expression of *LYVE1*, a marker for tissue-resident macrophages. This finding indicated that Mφ3 represents tissue-resident M2-polarized macrophages and displays high expression of *LYVE1*, a marker for tissue-resident macrophages, indicating that Mφ3 represents tissue-resident M2-polarized macrophages (42). When compared to that in uiMAT, Mφ1 in CrF showed an increase in the expression of genes related to phagocytosis, including “Positive regulation of phagocytosis” and “Regulation of phagocytosis.” The Mφ2 population was highly enriched in antigen-presenting pathways, such as “Antigen processing and presentation of peptide or polysaccharide antigen via MHC class II,” whereas Mφ3 showed an increase in metal metabolism-related signals, including “Transition metal ion homeostasis” (**Figure 2E**).

Next, we explored B cells and Plasma cell populations (**Figure 2F**). B cells, which features high expression of *CD74* and *MA4A1* (**Figures 2G, H**), displayed increased immunoactivity-related signatures, including “B cell activation” and “B cell receptor signaling pathway” in CrF compared to uiMAT (**Figure 2I**). Further subclustering of plasma cells identified distinct populations of IgG+ and IgA+ plasma cells (**Figure 2F**), with the former showing upregulation of genes encoding immunoglobulin G, including *IGHG2*, and the latter displaying an increase in genes encoding immunoglobulin A, including *IGHA2* (**Figures 2G, H**). Notably, IgG+ plasma cells in CrF exhibited enhanced signals related to complement system activation and phagocytosis, which suggests a role in the opsonization of foreign antigens through the complement system (43). Conversely, IgA+ plasma cells showed insignificant activity in CrF compared with that of uiMAT. We also subdivided T cells into four groups based on their gene expression and enriched pathways: Naïve T cells, characterized by the expression of *IL7R*+ and *CD3D*-; CD4 T cells, defined by *CD3D*+ and *CD4*+ expression; CD8 T cells, marked by *CD3D*+ and *CD8A*+ expression; and NK cells, identified by the expression of *NKG7*+, *GZMB*+, and *CD8A*- (**Figures S1A-D**). We observed no significant difference in immune activity among the T cell clusters in CrF, emphasizing the crucial role of macrophages, B cells, and plasma cells in the pathology of CrF. (Data not shown).

Collectively, our findings highlight CrF-specific immune cell activation and demonstrate the heterogeneity of macrophages, B cells, and plasma cell populations that drive the pathology of CrF.

### 3.3 CrF is characterized by an increase in committed PACs and their enhanced inflammatory response

**Figure 3:**
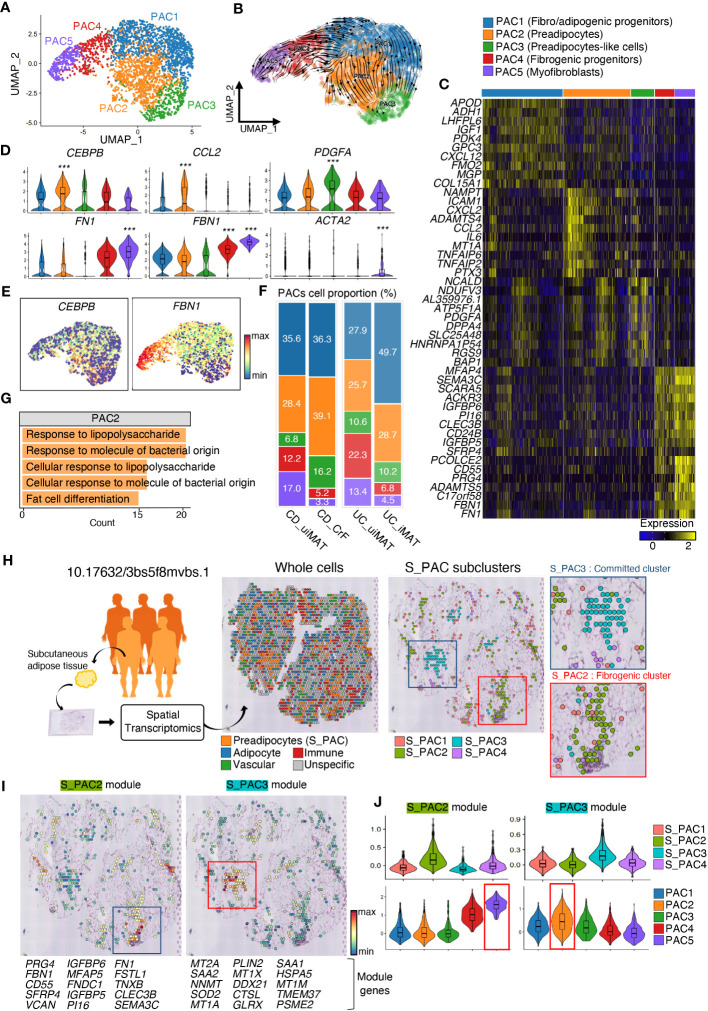
CrF Is Characterized by an Increase in Committed PACs and Their Enhanced Inflammatory Response. **(A)** UMAP plot shows the PACs isolated from **Figure 1E**, and the cluster analysis revealed five distinct clusters. **(B)** RNA-velocity analysis was performed on the PAC clusters, with the velocity field projected onto the UMAP plot from **(A)**. The arrows depict the local average velocity assessed on a regular grid, indicating the extrapolated future states of cells. **(C)** Distinct expression profiles of the three PAC subpopulations. **(D)** Violin plots showing the RNA expression levels of selected cluster markers for specific cell clusters **(E)** Feature plots depict the expression of CEBPB and FBN1 in PACs. **(F)** A bar plot showing the proportion of subclusters within a PAC cluster for each patient group. **(G)** Enriched Gene Ontology terms of the molecular signature for each subpopulation, hypergeometric test, adjusted a p < 0.01. **(H)** Schematic representation of the experimental procedure. Spatial transcriptomic data of subcutaneous adipose tissue from lean individuals (n = 3) and those with obesity (n = 5) were recruited from 10.17632/3bs5f8mvbs (left). The distribution of the overall cell clusters (middle) and subclusters of S_PACs (preadipocytes from the spatial transcriptomic data, right) is shown across an adipose tissue section of an obese individual. **(I)** Spatial representation of each module, consisting of the top 15 upregulated genes, for S_PAC2 and S_PAC3, respectively. **(J)** Violin plots showing the expression levels of each module in PACs from **Figure 4H** (top) and **(A)** (bottom). *** adjusted p < 0.001.

The PAC clusters were reclustered into five subclusters, namely PAC1, PAC2, PAC3, PAC4, and PAC5 (**Figure 3A**). Analysis of transcriptional dynamics and the differentiation process in PACs of CrF have been performed by RNA velocity analysis. The resulting vector field displayed two separate lineages of PACs, with PAC1 serving as the progenitor cluster, differentiating into a lineage leading to PAC2 and another lineage leading to PAC5 (**Figure 3B**). The molecular signatures of PAC2 and PAC5 differed greatly, implying heterogeneous differentiation of PACs (**Figures 3C–E**, **S2A**). The PAC2 cluster exhibited high expression of the *CEBPB* and *CEBPD* gene, which encodes the transcription factor C/EBP-β and C/EBP-δ that function in adipocyte differentiation (44). An increase in the proportion of PAC2 was observed in CrF compared with that in uiMAT, emphasizing the role of the PAC2 subcluster in CrF formation. The PAC2 cluster in iMAT was not significantly higher than that in uiMAT in patients with UC, demonstrating the significance of PAC2 in CrF (**Figure 3F**). The enriched pathways of the PAC2 cluster in CrF included the adipogenic pathway of “Fat cell differentiation” (**Figures 3G**, **S2B**). Interestingly, pathway analysis of the PAC2 cluster also showed multiple activated pathways related to bacterial infection, including “Response to lipopolysaccharide” and “Response to molecule of bacterial origin,” suggesting PAC2 as a PAC subpopulation that directly responds to bacterial infection. Another terminal type of PAC, the PAC5 cluster, highly expressed fibrotic genes, such as *FN1* and *FBN1*, and expressed *ACTA2*, a marker gene for myofibroblasts (**Figures 3C-E**, **S2A**) (45–47). These results suggest that PAC5 is a type of PAC involved in adipose tissue fibrosis. We compared the CrF dataset with a spatial transcriptomic dataset generated from subcutaneous adipose tissue (SAT) of lean individuals and those with obesity to evaluate the function of the two distinct lineages of PACs (**Figure 3H**). Within each PAC cluster in SAT, we identified unique sets of genes, called “modules,” that exhibited distinct expression patterns. These modules comprised the top 15 most highly upregulated genes in each cluster (**Figure 3I**). By analyzing the module scores in CrF PACs, we observed that the genetic signature of the PAC2 cluster closely resembles that of the committed cluster in SAT. On the other hands, the genetic signature of the PAC5 cluster exhibits the highest similarity to the fibrotic cluster in SAT (**Figure 3J**). These results support our finding that the two distinct lineages of PACs in CrF, as determined by RNA velocity estimation, have functional roles in adipogenesis and fibrosis.

### 3.4 Pro-inflammatory and fibrotic signatures increase in committed PACs in CrF

**Figure 4:**
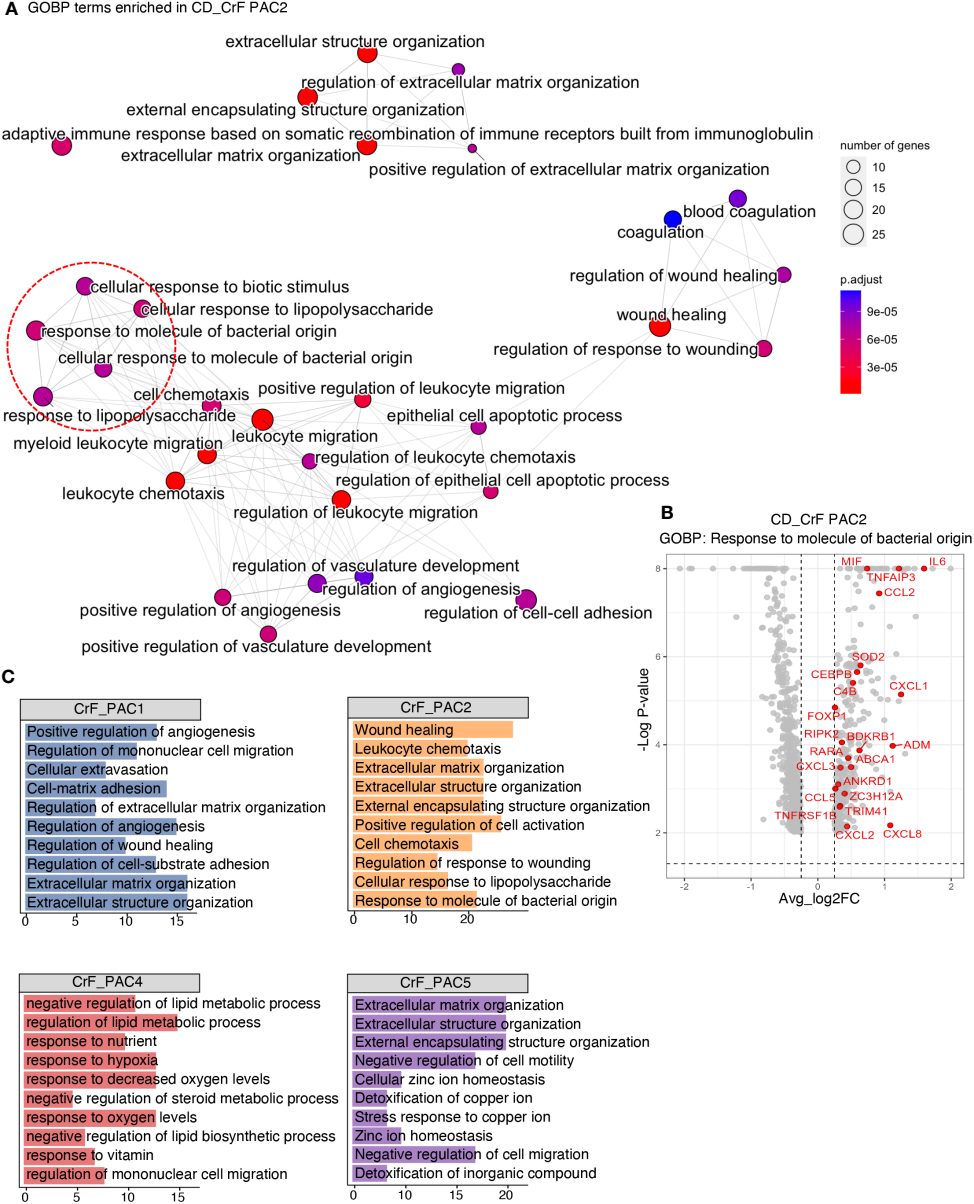
Pro-Inflammatory and Fibrotic Signatures Increase in Committed PACs in CrF. **(A)** ClusterProfiler revealed upregulated pathways of PAC2 in CD_CrF versus CD_uiMAT. Adjusted p < 0.05 was statistically significant. The pathways associated with the response to bacterial origin are indicated by red circles. **(B)** Volcano plot highlighting genes belonging to the pathway of Response to molecule of bacterial origin in up-regulated DEGs of PAC2 in CD_CrF versus CD_ uiMAT. **(C)** Enriched Gene Ontology terms of the molecular signature for each subpopulation in CD_CrF. Adjusted p < 0.01, hypergeometric test.

We explored the dysregulated pathways and identified enriched gene sets in PACs in CrF. Notably, pathways related to the response to the bacterial origin, such as “cellular response to biotic stimulus”, “cellular response to lipopolysaccharide”, “response to molecules of bacterial origin”, and “response to lipopolysaccharide”, were significantly upregulated in CrF compared to that in uiMAT in patients with CD (**Figure 4A**). However, no significant changes were observed in the iMAT of patients with UC. Our analysis revealed that the upregulated DEGs in PAC2, related to the “response to molecule of bacterial origin” included various cytokine genes, such as *MIF*, *IL6*, *TNFAIP3*, and *CCL2* (**Figure 4B**). Preadipocytes respond to bacterial infections by producing and releasing proinflammatory cytokines such as *TNF-α*, *IL-6*, and *IL-8*, which trigger inflammation and attract immune cells to the affected area (48, 49). Once activated, these immune cells also secrete cytokines and other inflammatory molecules, amplifying the overall inflammatory response in the local and systemic tissues. Therefore, our results suggest that PAC2 contributes significantly to the inflammatory response in CrF. We found that pathways related to fibrosis, including “extracellular matrix remodeling” and “extracellular component remodeling,” were significantly enriched in CrF (**Figure 4C**). These pathways were broadly enriched not only in PAC2 but also in other subtypes of PACs, including the highly fibrogenic PAC5 in CrF. Studies indicate increased fibrosis and inflammation in the adipose tissues of obese individuals (50–52). To better understand the characteristics of PACs in CrF, we analyzed single nucleus RNA sequencing data from the visceral adipose tissue (VAT) of obese individuals (BMI 40-50) (**Figure S3A**) (27). In comparison to PACs in CrF, obese individuals showed a decrease in the enrichment score for fat cell differentiation and gene expression related to insulin reactivity and lipid storage (**Figure S3B**). No significant differences were found in the pathways associated with the inflammatory response. However, similar to CrF, obese individuals exhibited an elevated enrichment score for pathways related to extracellular matrix organization.

### 3.5 Fibrotic PACs in both CD patients and lymphedema patients exhibit transcriptional similarities

**Figure 5:**
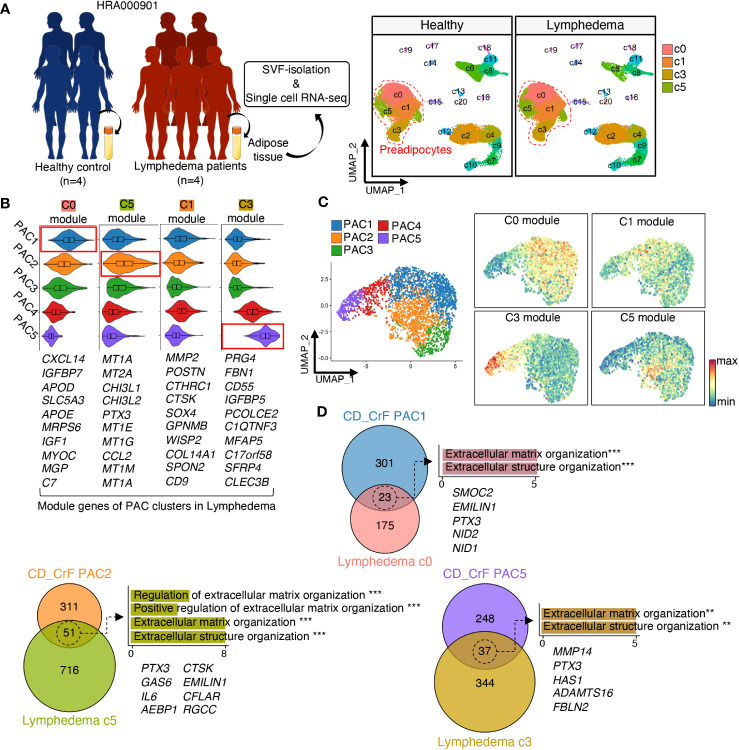
Fibrotic PACs in Both CD Patients and Lymphedema Patients Exhibit Transcriptional Similarities. **(A)** Schematic representation of the experimental procedure. Data of patients with cancer-related lymphedema (n = 5) and healthy individuals (n = 4) were recruited from HRA000901 (left). UMAP revealed 21 distinct cellular clusters of 70209 cells, of which c0, c1, c3, and c5 were identified as preadipocytes based on the expression of established marker genes. (right) **(B, C)** The expression levels of each module, which consisted of the top 10 upregulated genes in each PAC subcluster from **(A)**, were analyzed in preadipocytes from **Figure 3A**. **(D)** The Venn diagram illustrated the number of overlapping DEGs between similar preadipocyte cell clusters in CD_CrF and lymphedema data. The enriched Gene Ontology terms related to fibrosis were analyzed using the overlapping DEGs, and the genes belonging to these pathways are shown. ** adjusted p < 0.01; *** adjusted p < 0.001

Next, we compared the gene signatures of PACs in CrF with those of PACs in subcutaneous adipose tissue in patients with lymphedema (**Figures 5A**, **S4A, B**). Lymphedema is a chronic condition that occurs when the lymphatic flow is blocked, leading to the expansion of adipose tissue and fibrosis around lymphatic vessels in response to injury or bacterial infection (53). As CrF is rich in lymph nodes, there may be similarities between the changes observed in adipose tissue in lymphedema and those found in CrF (54, 55). This similarity could potentially provide insights into the mechanisms underlying adipose tissue changes under these conditions. First, we identified modules consisting of the top 10 marker genes for each PAC cluster in adipose tissues from patients with lymphedema. After scoring CrF PAC clusters using the module of the top 10 marker genes in lymphedema, we observed that PAC1 exhibited a gene expression signature most similar to PAC cluster c0, while PAC2 showed the highest similarity to PAC cluster c5. On the other hand, PAC5 had the closest resemblance to lymphedema cluster c3, which is known as the primary contributor to fibrosis (**Figures 5B, C**). Our analysis indicated that unlike CrF PAC2, the lymphedema c5 cluster did not display significant changes in bacterial response or inflammation-related pathways. Rather, we observed an increase in fat cell differentiation of c5 and an up-regulation in the extracellular matrix remodeling pathway in the overall PAC, similar to what was found in CrF PAC (**Figure S5A**). These findings suggest that while inflammatory changes in lymphedema and CrF may differ, both conditions are characterized by alterations in pathways related to fibrosis. To identify key genes related to fibrosis, we compared the gene signatures of PACs in CrF and lymphedema adipose tissues and identified shared upregulated genes (**Figure 5D**). Subsequently, we conducted a GO enrichment analysis of these genes, which revealed pathways related to fibrosis, with Pentraxin3 (*PTX3*) being a common gene in both conditions. The *PTX3* gene has been implicated in fibrotic diseases, such as pulmonary and renal fibrosis, and its inhibition reduced fibrosis in a mouse model (56, 57). These results suggest that *PTX3* may play a significant role in fibrosis. In the context of obesity, the expression of *PTX3* in preadipocytes within VAT has been observed to be rare (**Figure S3C**). Additionally, our investigation of genes associated with extracellular matrix organization pathways, which are commonly upregulated in both obesity and CrF, revealed no significant upregulation comparable to the expression levels of *PTX3* observed in CrF and lymphedema.

### 3.6 PACs play a key role in distinctive cell-to-cell communication in CrF

**Figure 6:**
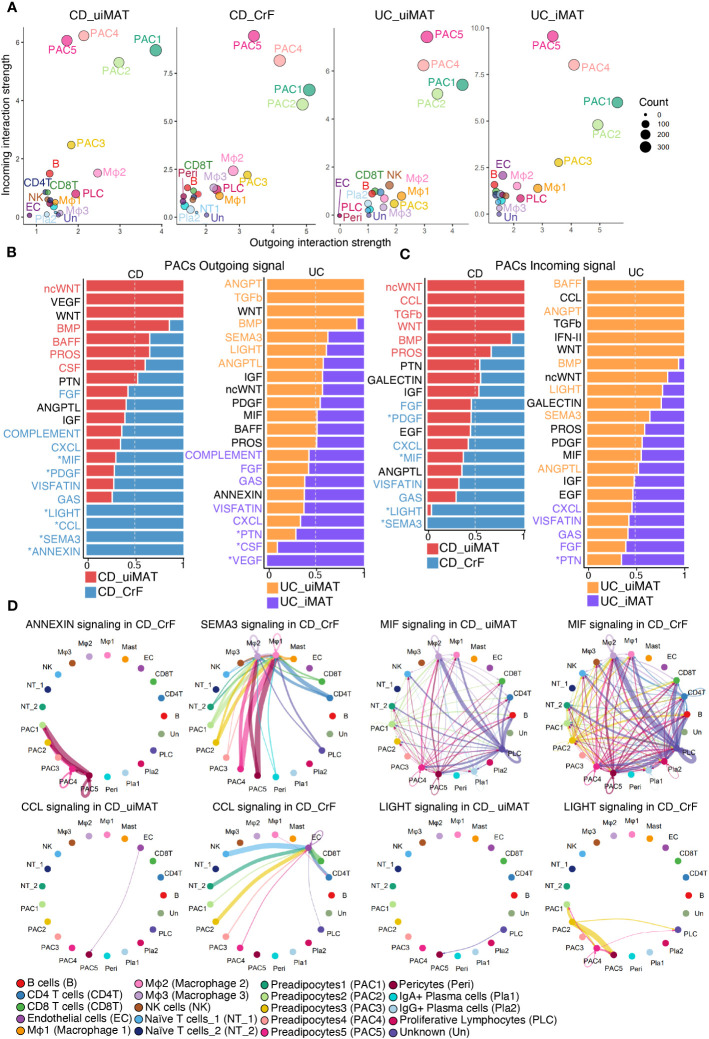
PACs Play a Key Role in Distinctive Cell-to-Cell Communication in CrF. **(A)** Scatter plots showing the strength of outgoing and incoming interactions, enabling identification of the cell populations exhibiting significant changes in sending or receiving signals. **(B)** Bar plots showing the ranking of outgoing signals of PACs in CD_CrF versus CD_uiMAT (left) and UC_iMAT versus UC_uiMAT (right). The ranking of signals was determined based on differences in the strength of information flow, calculated as the sum of communication probabilities among all pairs of cell groups in the inferred network. **(C)** Bar plots showing the ranking of incoming signals of PACs in CD_CrF versus CD_uiMAT (left) and UC_iMAT versus UC_uiMAT (right). **(D)** Circle plots showing the inferred signaling network upregulated in CD_CrF. The arrows and edge color represent the direction (source: target). The edge colors are consistent with the sources as sender, and edge weights are proportional to the interaction strength. Thicker edge line indicates a stronger signal.

CellChat was employed to investigate changes in cell-to-cell communication in CrF. PACs showed the highest interaction in both CrF from patients with CD and iMAT from patients with UC (**Figures 6A**, **S6A**). First, we analyzed the outgoing signaling, which refers to signals emitted by a cell to influence neighboring cells, from PACs. We confirmed the increased signaling of PACs in CrF compared to uiMAT from patients with CD, as well as in iMAT compared to uiMAT from patients with UC (**Figures 6B, D**, **S6B**). Next, we compared the upregulated interaction in CrF from patients with CD and iMAT from patients with UC. PACs in CrF specifically exhibited an increase in signaling pathways such as, LIGHT, CCL, SEMA3, and ANNEXIN, whereas PACs in iMAT from patients with UC showed an increase in PTN, CSF, and VEGF signaling pathways. We explored incoming signals, which represent signals received by neighboring cells, in PACs. We found enriched PDGF, MIF, LIGHT, and SEMA3 signaling pathways in PACs from patients with CD, while PTN was enriched in iMAT from patients with UC (**Figures 6C, D**, **S6C**). Interestingly, in cases of lymphedema, all prominent outgoing signals from the PAC in CrF, including LIGHT, CCL, SEMA3, and ANNEXIN, showed an increase. Similarly, the incoming signals such as PDGF, LIGHT, and SEMA3 to the PAC in CrF also exhibited an increase, with the exception of MIF. (**Figures S7A, B**). On the other hand, in the case of obesity, only SEMA3 among the outgoing signals in PACs showed an increase (**Figures S8A, B**). In various tissues, including adipose tissue, PDGF signaling is crucial for fibrosis development because it promotes the proliferation and migration of fibroblasts and their production of excessive extracellular matrix (58, 59). For instance, high-fat diet-induced fibrosis in adipose tissue is associated with increased PDGF upregulation and fibrosis-related gene expression (60). *In vitro* studies have confirmed that PDGF induces adipose-derived stem cell differentiation into myofibroblasts (61). Consistently, our data showed that PAC3, characterized by high PDGFA expression, was strikingly increased in CrF compared to that in other PACs in patients with CD. Therefore, the role of PDGF-releasing PAC3 may be important for the activation of PACs involved in CrF fibrosis and consequently in the fibrostenosis of CD. The LIGHT signaling contributes to fibrosis by activating fibroblasts, stimulating extracellular matrix production, and triggering the release of proinflammatory cytokines in PACs, leading to metabolic dysfunction (62–64). The pro-inflammatory cytokine, MIF, causes adipose tissue dysfunction, leading to obesity by promoting the release of pro-inflammatory cytokines, adipocyte differentiation, and immune cell infiltration and activation, resulting in inflammation and metabolic dysfunction (65–67).

### 3.7 High fibrotic CrF exhibits increased IgG+ plasma cells and pentraxin-3 expression in CD patients

**Figure 7:**
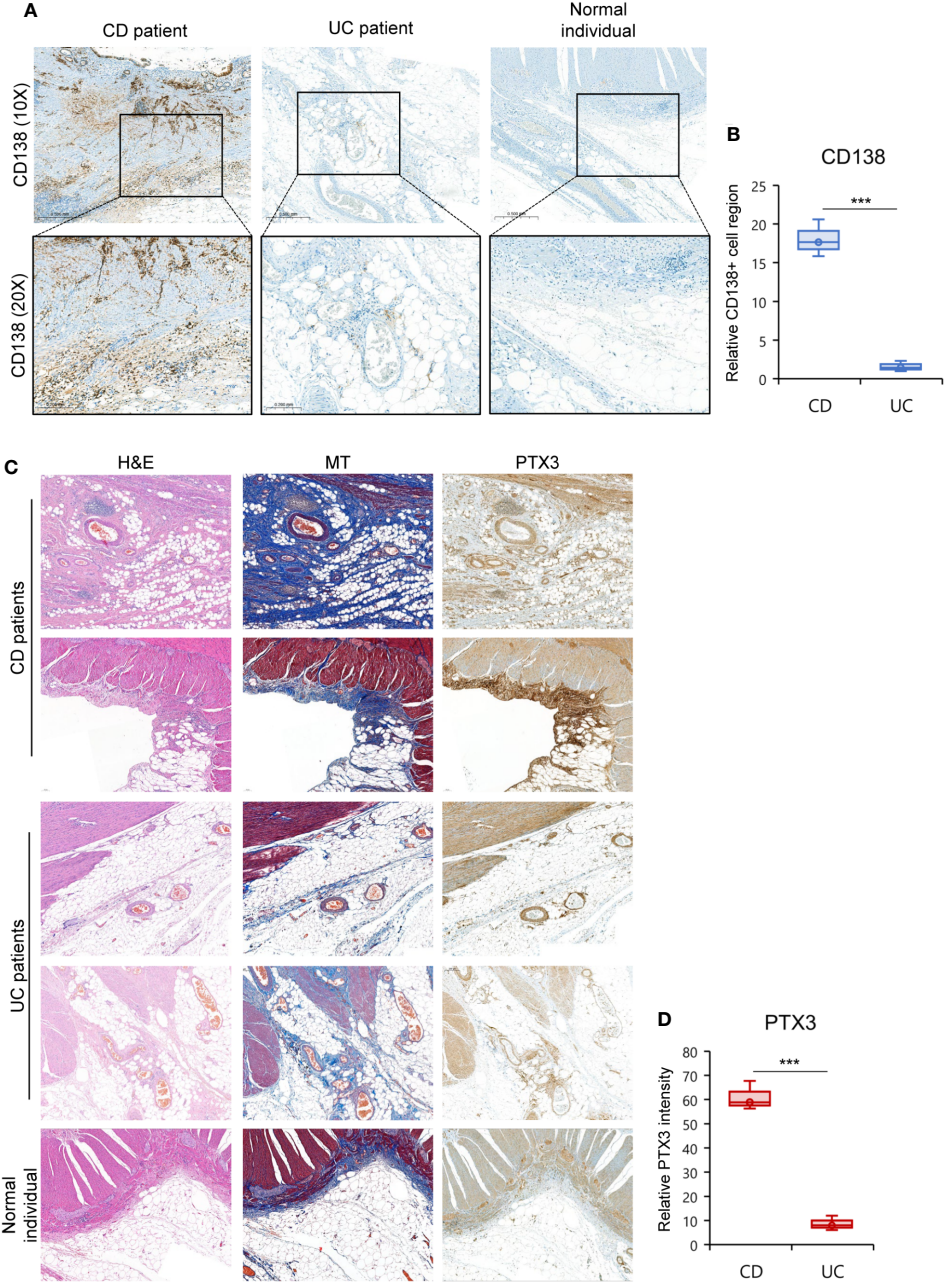
High Fibrotic CrF Exhibits Increased IgG+ Plasma Cells and Pentraxin-3 Expression in CD Patients. **(A, D)** Representative images for histopathological evaluation of CrF in CD patients (n=3), iMAT in UC patients (n=3), and MAT from a normal individual (n=1). **(A)** IgG+ plasma cells were stained with CD138 (syndecan-1). **(B)** Densitometry analysis of CD138+ cells in (**A**, **Figure S9**). Box plots compare the relative CD138+ cell regions in CD and UC patient samples to those in the normal sample. **(C)** Hematoxylin-eosin (H&E) and masson trichrome (MT, staining tissue fibers), and pentraxin-3 (PTX3) staining was presented separately. **(D)** Box plots compare the relative PTX3 intensity in CD and UC patient samples to those in the normal sample. *** p < 0. 001; two-tailed t test.

To substantiate our findings at the protein level, we conducted immunohistochemistry staining on the CrF of CD patients, comparing it to MAT from UC patients and normal individuals. We found a significant increase in the infiltration of IgG+ plasma cells (CD138+ cells), particularly within the CrF (**Figures 7A, B**, **S9**). Furthermore, we identified substantial fibrosis within the CrF, accompanied by a noteworthy increase in pentraxin-3 expression within highly fibrotic regions. In contrast, MAT from UC patients and normal individuals rarely exhibited pentraxin-3 expression (**Figures 7C, D**, **S9**).

**Figure 8:**
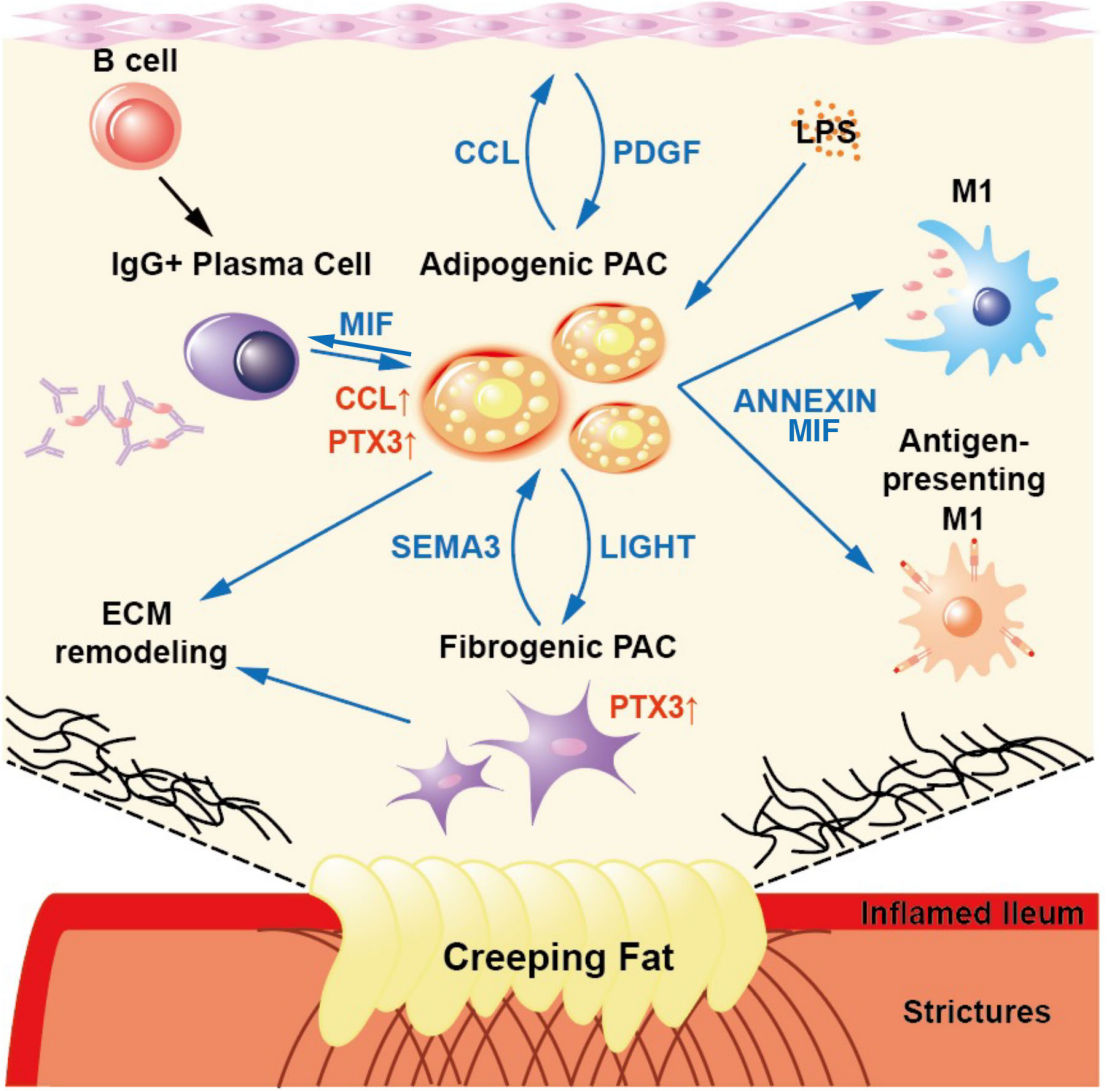
A Schematic Showing the Results of This Study. The committed PACs in CrF demonstrated both pro-inflammatory and pro-fibrotic activity, as well as specific cell-to-cell interactions within CrF.

In summary, we identified CrF-associated cell subpopulations, particularly PACs, as well as immune cells, including pro-inflammatory macrophages, B cells, and IgG+ plasma cells. By comparing our results with spatial transcriptomic adipose tissue data, single nucleus RNA-seq data of obese individuals, and scRNA-seq data of adipose tissue from patients with lymphedema, we identified specific genetic features of PACs related to fibrosis and inflammation in CrF (**Figure S10**). The unique characteristics observed in CrF was a distinct high inflammatory response via committed PAC, with an enriched MIF signaling pathway via PACs. Also, we found the similarities between PACs in CrF and lymphedema in fibro-genic features with pentraxin-3 expression and cell-to-cell interactions (**Figure 8**). Finally, by demonstrating increased pentraxin-3 expression at the protein level within the fibrotic CrF in CD patients, our study highlights pentraxin-3’s potential as a novel target for treating CrF fibrosis.

## 4 Discussion

A comprehensive understanding of cellular heterogeneity and regulatory modifications in affected tissues is essential for developing effective remedies for CD. One potential target of CD is CrF, a unique and significant feature found only in patients with CD (68, 69). The creeping fat, a layer of visceral adipose tissue, surrounds the intestine in patients with CD and is characterized by the infiltration of immune cells and fibrosis (70). This infiltration results in the production of proinflammatory cytokines and chemokines, leading to chronic inflammation and tissue damage. Moreover, the inflammatory cells present in CrF can migrate to the inflamed intestinal mucosa, further exacerbating inflammation and tissue injury (71). To gain a deeper understanding of the cellular and molecular mechanisms of CrF, we analyzed scRNA-seq data from CrF and adjacent uiMAT in patients with CD and compared it to MAT from the adjacent inflamed and uninflamed intestines of patients with UC. Using an unbiased clustering approach, we identified 18 cell clusters and assigned them to 10 distinct cell lineages. We further analyzed PACs that showed the most different transcriptomic features in CrF. By sub-clustering PACs and calculating their velocity, we identified a two-pronged pathway for their development into myofibroblasts or adipocytes. The PAC2 cluster represented the cluster in the most committed stage during adipogenesis, with high expression of *CEBPB* and genes related to fat cell differentiation. The PAC5 cluster, however, was the most similar cluster to myofibroblasts, with high expression of marker genes, such as *FN1*, *FBN1*, and genes related to fibrosis. Additionally, by comparing our data to spatial transcriptomic data of adipose tissue, we confirmed that the PAC cluster with genetic similarities to PAC2 underwent the adipogenesis stage and was found in adipocyte-containing areas. In contrast, the PAC cluster with genetic similarities to PAC5 was in high-fibrosis regions of adipose tissue, indicating its potential role in the fibrosis pathway. Furthermore, we observed a significant increase in the proportion of PAC2 among the five sub-clusters in CrF in patients with CD, whereas no such increase was observed in iMAT in patients with UC. Notably, this cluster was highly responsive to bacterial stimuli, as evidenced by the upregulated expression of pro-inflammatory cytokine genes, whereas other disease condition including iMAT in patients with UC, subcutaneous adipose tissue from patients with lymphedema, and VAT in obese individual not showed inflammatory activity. A microbiome study demonstrated that bacterial species, particularly *Clostridium innocuum*, can translocate to MAT and promote adipogenesis in a gnotobiotic mouse with a simplified microbiota. These results suggest that bacterial stimulation may contribute significantly to the enhanced adipogenesis and inflammatory capacity of PACs in CrF. In addition to immune cells, PACs with increased inflammatory activity in CrF, could exacerbate inflammation in the adjacent intestinal tissue in patients with CD.

Fibrosis is a major contributor to the pathology of CD, particularly in the development of strictures (72). The accumulation of fibrotic tissue in CrF can lead to the narrowing of the intestinal lumen and obstruction, as well as distortion and thickening of the intestinal wall, which can lead to inflammation and further tissue damage (73). In our study, we found that PAC5, along with PAC2 and PAC3, were significantly enriched in the fibrosis pathway, including “extracellular matrix organization” and “collagen formation,” indicating the severity of fibrosis in CrF. To identify potential target genes for alleviating fibrosis in CrF, we compared the gene expression of PACs from adipose tissue between lymphedema and CrF. Our findings revealed that *PTX3* was the most highly overlapping gene, suggesting that it may be a promising target for reducing fibrosis in CrF. In the case of VAT in obese individuals, there was an increase in the expression of genes related to extracellular matrix organization; however, the expression of *PTX3* was barely observed. This suggests that the up-regulated expression of *PTX3* is a distinct characteristic of creeping fat and fibrotic adipose tissue in lymphedema. As revealed in other study (19), pro-inflammatory roles of macrophages are significantly increased in CrF, such as cytokine secretion, phagocytosis, and antigen presentation. Moreover, we found that B and IgG+ plasma cells showed increased immunological activity in CrF. B-cell activation is important for generating specific antibodies that can neutralize pathogens and protect the body against infections. The IgG+ plasma cells, which produce IgG isotype antibodies, play a critical role in systemic immunity. These antibodies provide long-lasting protection against pathogens by binding to them and promoting their clearance by immune cells such as macrophages and natural killer cells (74, 75). Additionally, IgG antibodies activate the complement system, which directly kills pathogens and amplifies their immune response (76). Our findings suggest that immunological tissue dysfunction may contribute to the pathophysiology of CrF. Furthermore, our investigation of intercellular communication in CrF using CellChat revealed significant changes, with PACs displaying the highest levels of interaction in both patient derived CrF and iMAT from patients with CD and UC, respectively. We identified specific signaling pathways enriched in PACs in CrF, including CCL, ANNEXIN, LIGHT, PDGF, MIF, and SEMA3 pathways. These pathways may play a role in the recruitment and activation of immune cells, particularly endothelial cells, in response to inflammation, potentially contributing to the development of fibrosis and metabolic dysfunction in adipose tissue. Interestingly, there is a significant overlap in the patterns of increased interactions observed in perivascular adipocyte progenitor cells (PACs) between lymphoma and CrF. However, the MIF signaling pathway is unique to PACs in CrF.

In summary, our study has yielded novel findings that extend beyond previous single-cell studies. First, we identified two distinct PAC lineages in CrF, each following a two-pronged pathway towards adipocyte and myofibroblast differentiation. Notably, we observed that committed PACs in CrF exhibited pro-inflammatory activity and displayed increased MIF signals, which distinguish them from adipose tissue in other disease conditions. Furthermore, we identified significant similarities in the fibrotic features of PACs between CrF and SAT in lymphedema, including the up-regulation of *PTX3*, a fibrosis-associated marker, as well as specific cell-to-cell interactions involving ANNEXIN, LIGHT, CCL, and PDGF.

There still exists a dearth of well-designed randomized controlled trials specifically focusing on the resection of CrF. Furthermore, the standardization of CrF excision as a surgical procedure has not been achieved (77). Therefore, ongoing studies are dedicated to further investigating and addressing this subject. Notably, a significant finding from a single-center cohort study indicated that performing concurrent mesenteric excision during surgery led to a reduced risk of recurrence in patients with CD (78). Additionally, extensive mesenteric excision at the time of surgery was found to be an effective approach in lowering the risk factors for reoperation compared to limited mesenteric excision in CD cases (79). In this context, our research on the characteristics of CrF has promising potential as a valuable resource for clinical studies exploring the link between the presence of Creeping fat and the disease-free survival of individuals with CD.

Our study has several limitations. Firstly, conducting experimental validation *in vitro* or *in vivo* is necessary to confirm our findings. Specifically, further studies are needed to establish the relationship between *PTX3* expression and the fibrotic phenotypes observed in CrF. Additionally, investigating the upstream cell-to-cell interactions that influence the upregulation of *PTX3* would enhance our understanding of the underlying mechanisms. Finally, as the stromal vascular fraction in this study’s single-cell dataset excluded mature adipocytes, it is imperative to conduct further investigations to explore the genetic characteristics of the overall cell composition, including adipocytes, in CrF.

## Data availability statement

The datasets presented in this study can be found in online repositories. The names of the repository/repositories and accession number(s) can be found in the article/**Supplementary Material**.

## Ethics statement

The studies involving humans were approved by Yonsei University Gangnam Severance Hospital, Institutional Review Board. The studies were conducted in accordance with the local legislation and institutional requirements. The participants provided their written informed consent to participate in this study.

## Author contributions

NH and DK conducted most of the research and drafted the manuscript. SF designed this study. J-WK and JC were involved in the conceptualization of the study. S-JS, BY, J-WK, JC, and SF were primarily involved in data collection, hypothesis development, and manuscript development. All authors contributed to the article and approved the submitted version.
