# Supplementary material for: Creeping fat exhibits distinct Inflammation-specific adipogenic preadipocytes in Crohn’s disease
Source: Front Immunol. 2023 Dec 4;14:1198905. doi: 10.3389/fimmu.2023.1198905 (PMC10725931; doi:10.3389/fimmu.2023.1198905)
Supplement: Supplementary file 1 [file DataSheet_1.pdf]

Supplementary Material

1 Supplementary Figures

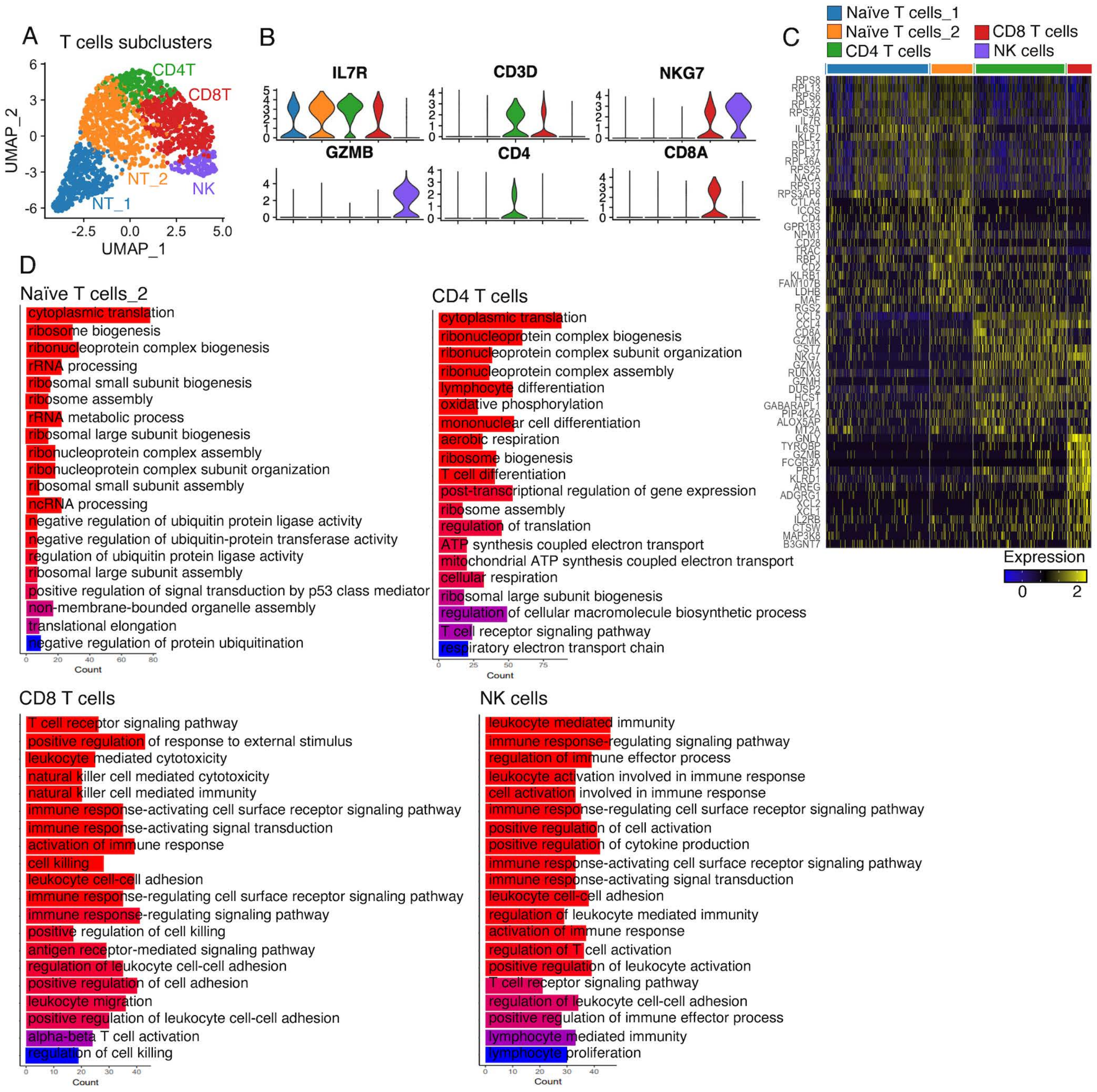

Supplementary Figure 1. Analysis of differentially expressed genes reveals distinct transcriptomic characteristics of T cell subclusters in creeping fat. **(A)** UMAP plot shows the T cells isolated from Fig. 1E, and the cluster analysis revealed three distinct clusters. **(B)** Violin plots showing the RNA expression levels of selected cluster markers for specific cell clusters. **(C)** Distinct expression profiles of the three subpopulations of T cells. **(D)** Enriched Gene Ontology terms of the molecular signature for each subpopulation. Adjusted P value < 0.01, the hypergeometric test.

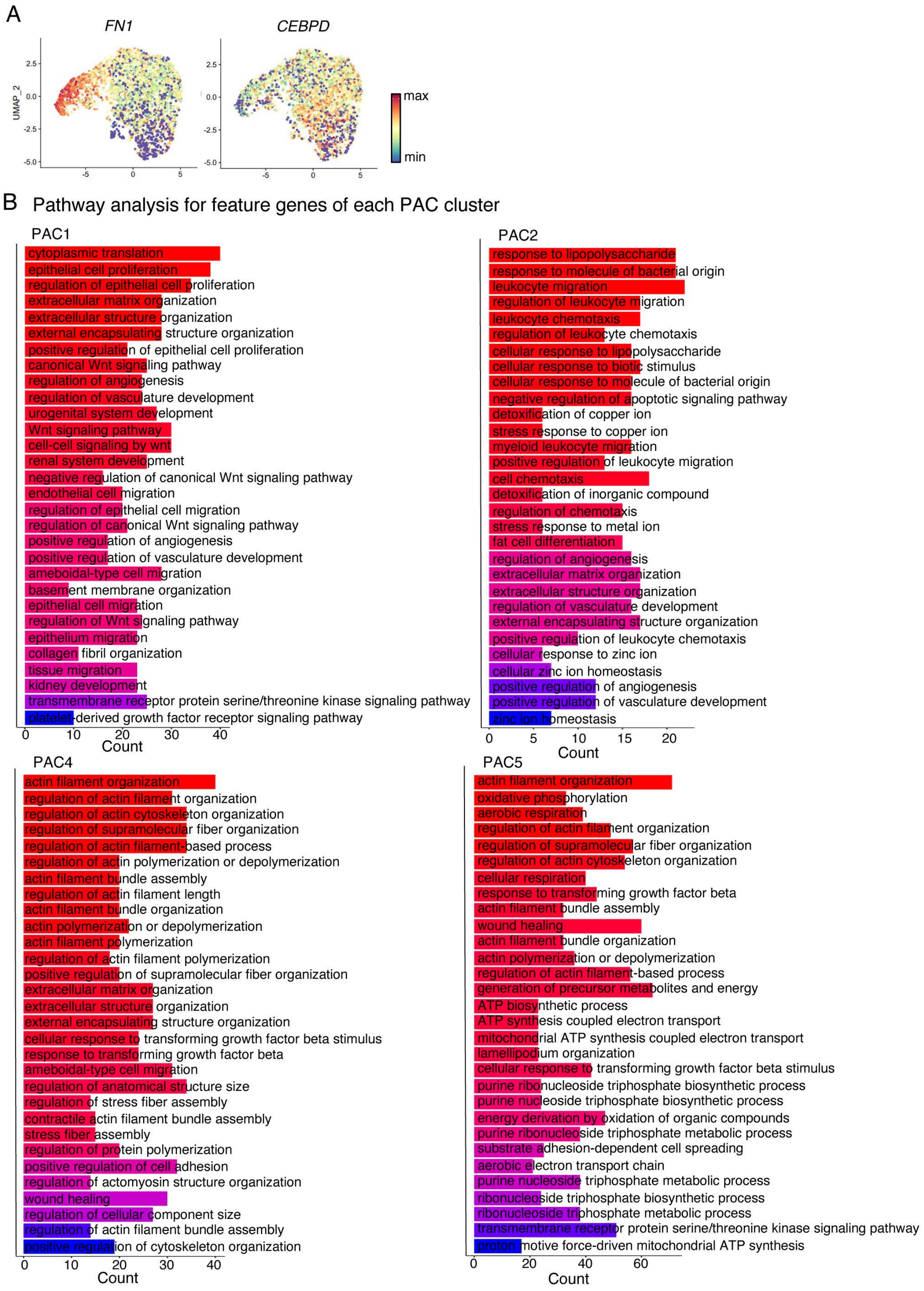

Supplementary Figure 2. Features of each preadipocyte subcluster in creeping fat **(A)** Feature plots depict the expression of *FN1* and *CEBPB* in PACs. **(B)** Enriched Gene Ontology terms of the molecular signature for each subpopulation. Adjusted P value < 0.01, the hypergeometric test.

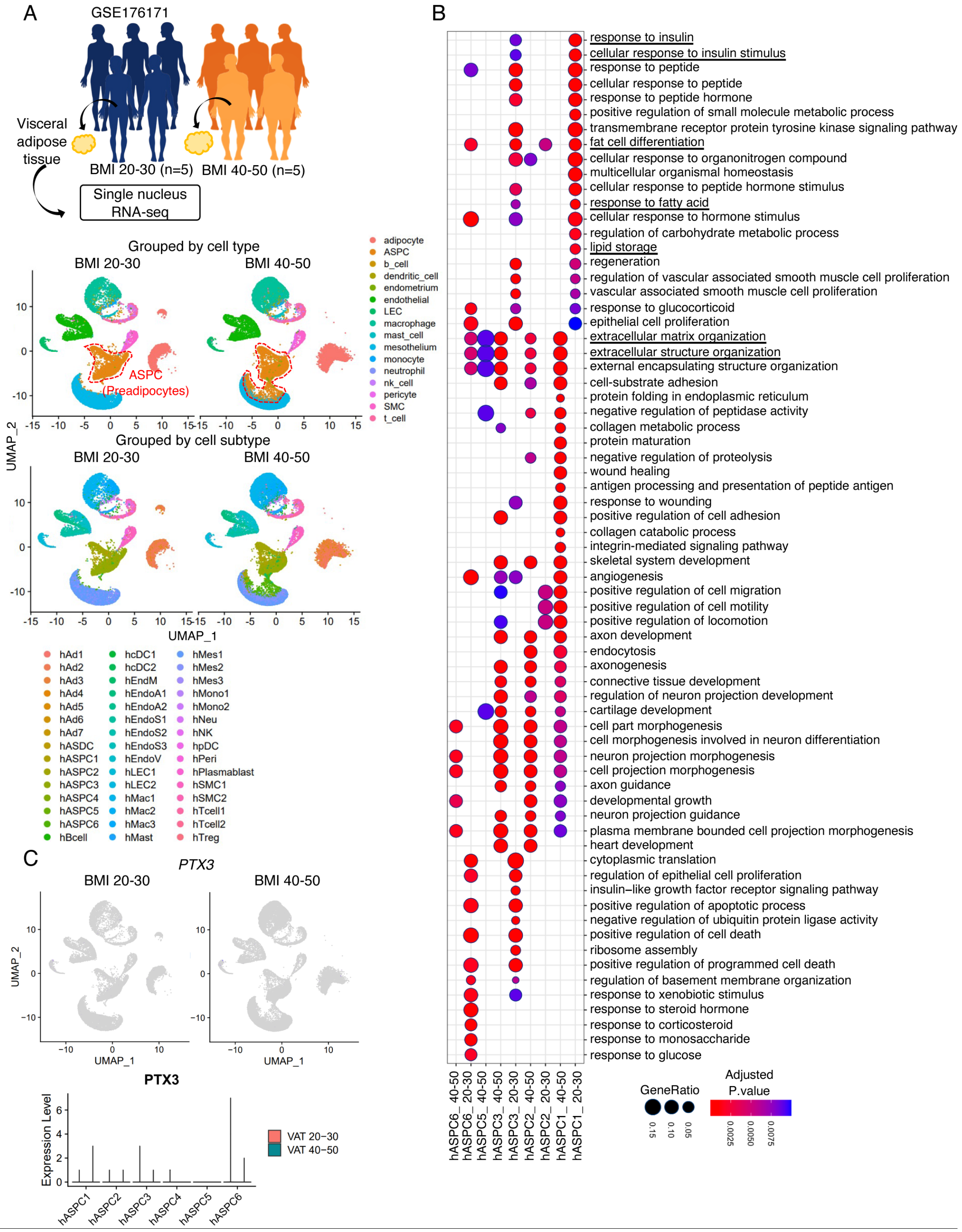

Supplementary Figure 3. The transcriptomic features of preadipocytes in visceral adipose tissue in obese individuals. **(A)** Schematic representation of the experimental procedure. Data of overweight (BMI 20-30, n = 5) and obese individuals (BMI 40-50, n = 5) were recruited from GSE176171(top). UMAP revealed 16 distinct cellular clusters (45 cellular subclusters). **(B)** Dot plot showing enriched Gene Ontology terms of the molecular signature for each adipose stem and progenitor cells (ASPC) subpopulation. Adjusted P value < 0.01, as determined by the hypergeometric test. **(C)** Feature plots and a violin plot depict the expression of *PTX3*.

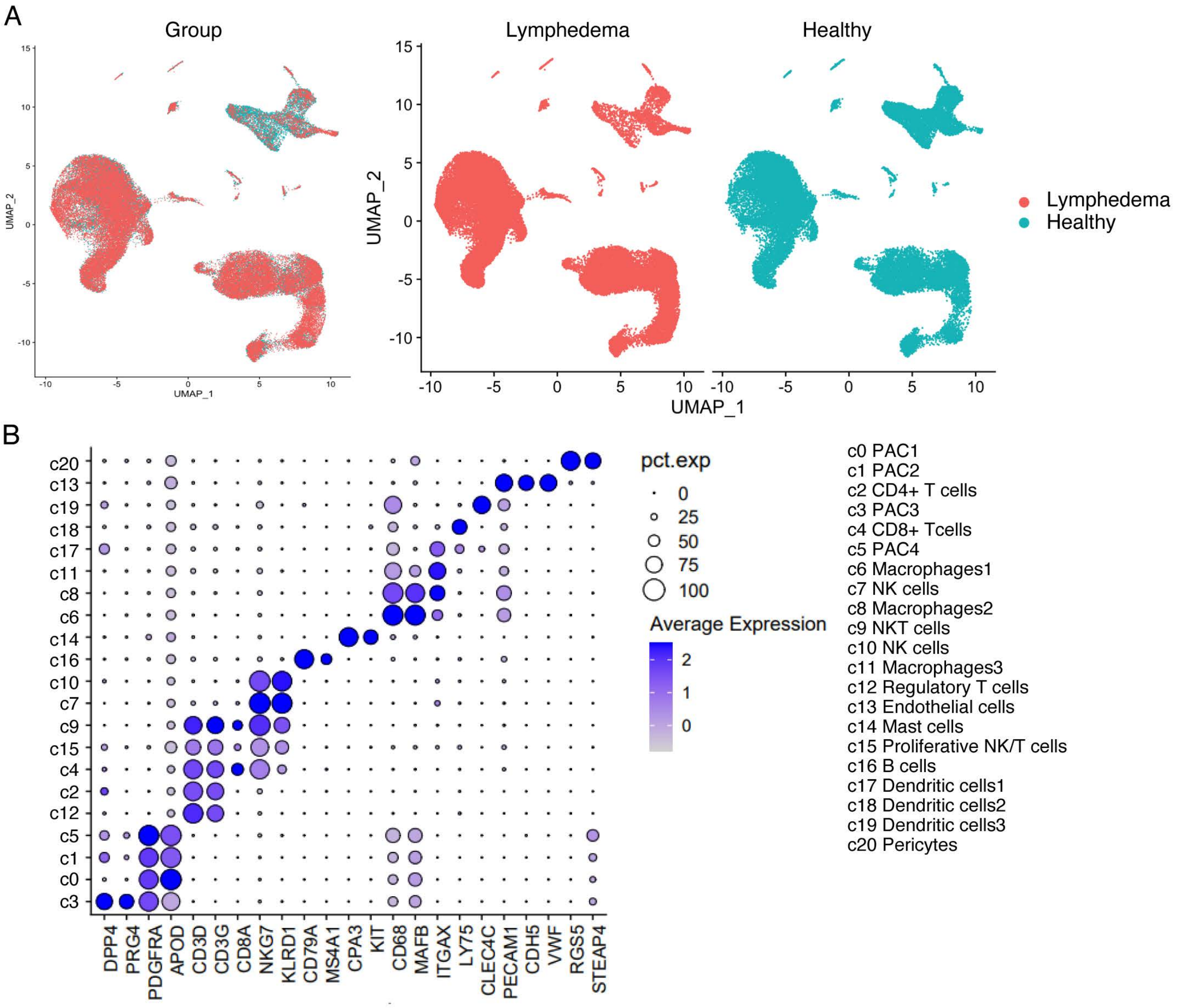

Supplementary Figure 4. Annotation of cell types in single-cell RNA data of lymphedema adipose tissue. **(A)** UMAP plot shows two distinct groups of adipose tissue data from healthy individuals and patients with lymphedema. The two distinct groups are colored in blue and red, representing healthy individuals and patients with lymphedema, respectively. **(B)** Dot plots and feature plots are used to visualize the expression of established marker genes for each lineage in each cluster.

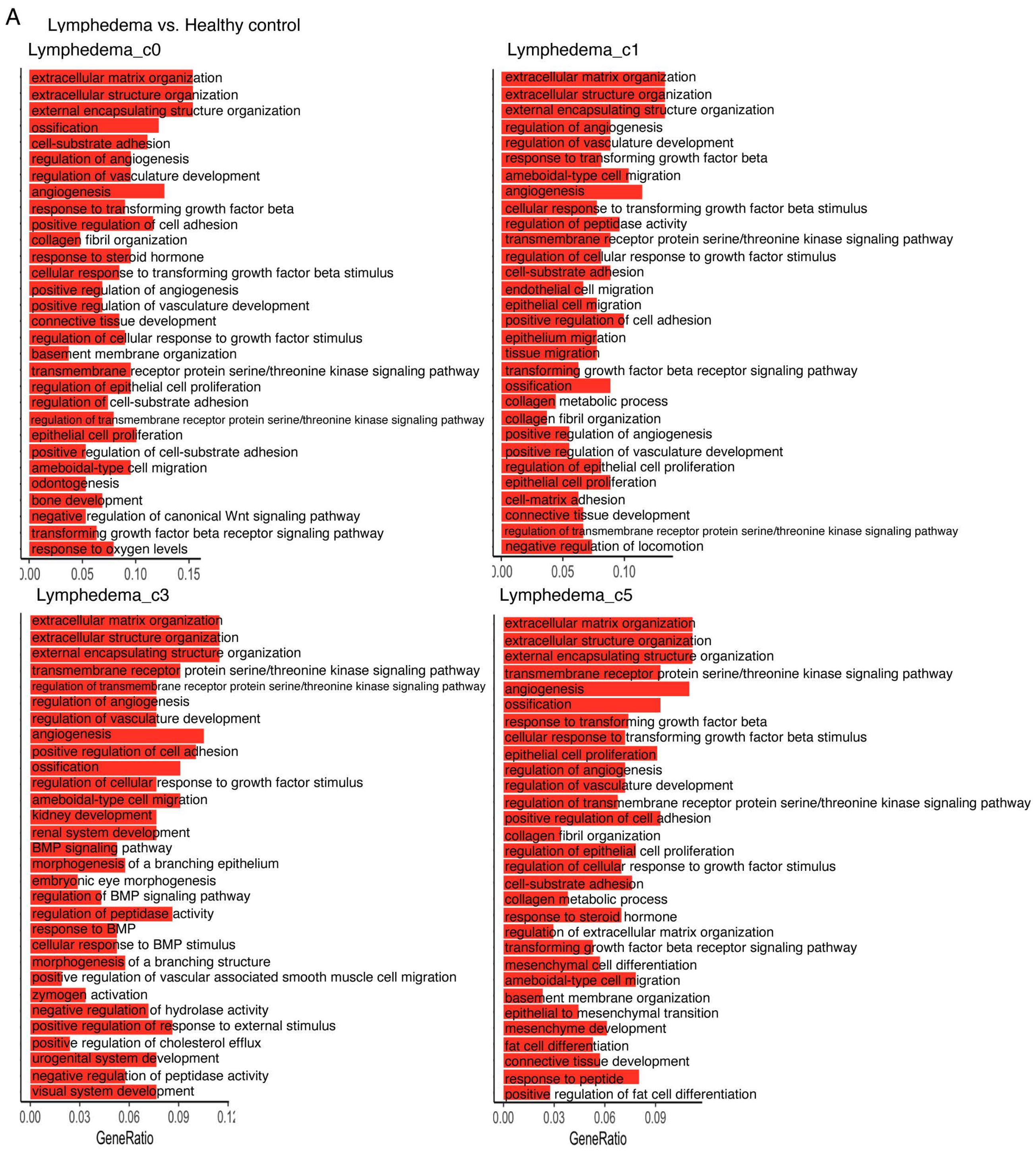

Supplementary Figure 5. Transcriptomic features of each preadipocyte subcluster in lymphedema adipose tissue. **(A)** Enriched Gene Ontology terms of the molecular signature for each subpopulation of PAC in adipose tissue from patients with lymphedema. Adjusted P value < 0.01, as determined by the hypergeometric test.

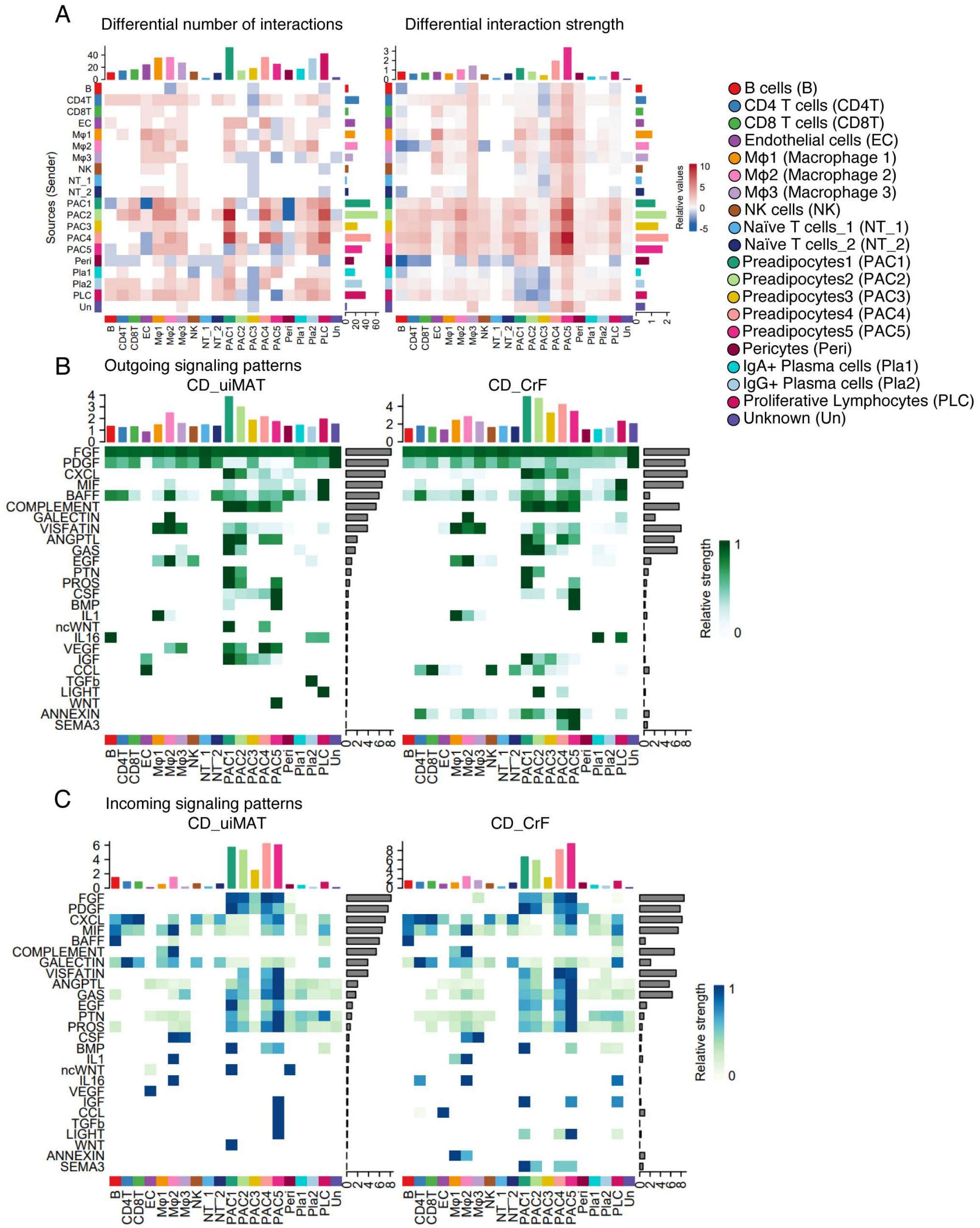

Supplementary Figure 6. Overall signals of cells in creeing fat in patients with crohn's disease. **(A)** Heatmap shows the differential number of interactions and differential interaction strength between CD_CrF and CD_uiMAT. In the color bar, red (or blue) represents increased (or decreased) signaling in the second dataset compared to the first one. **(B and C)** Heatmaps show the Outgoing **(B)** and Incoming **(C)** signaling patterns between CD_CrF and CD_uiMAT.

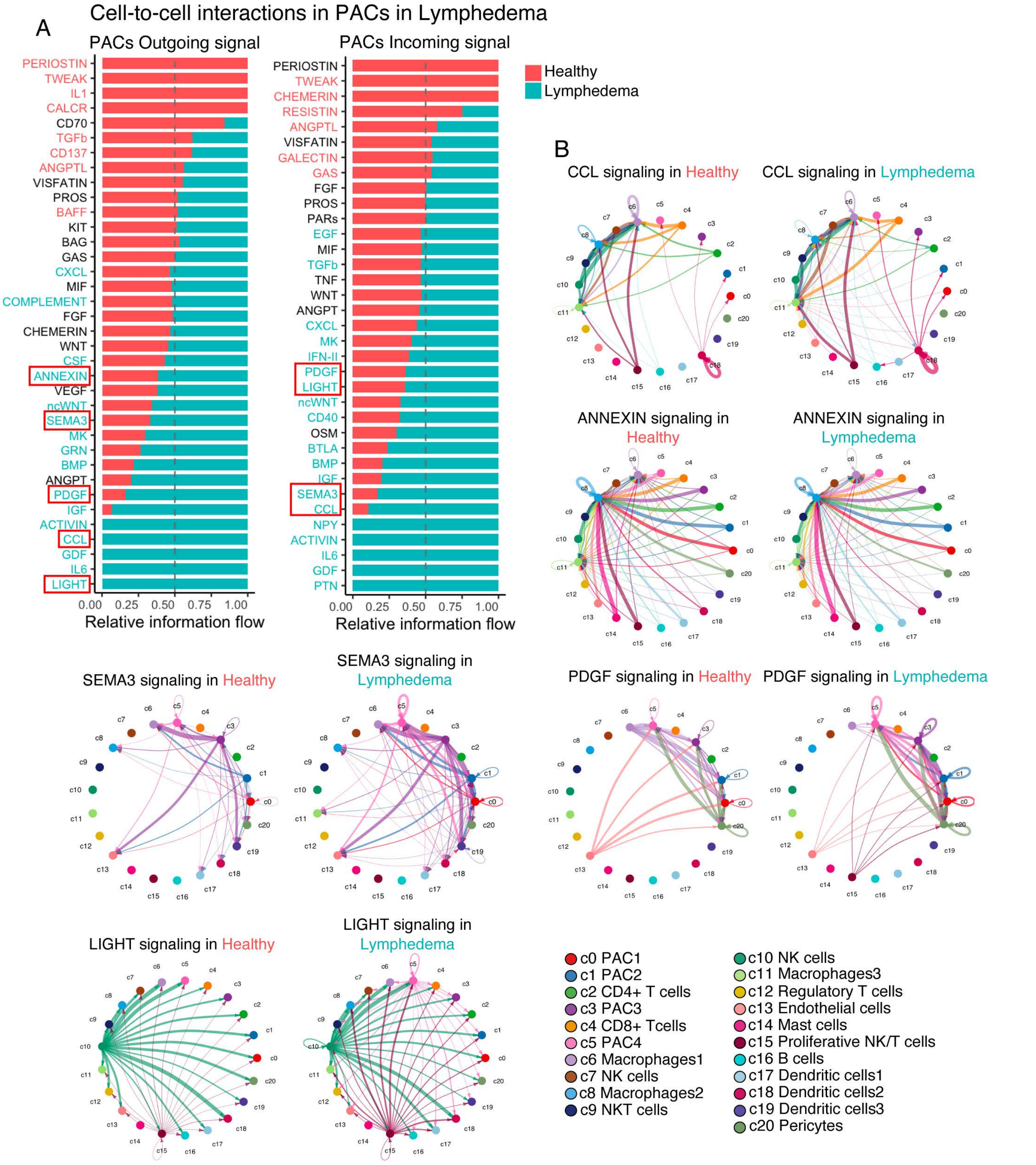

Supplementary Figure 7. Analysis of cell-cell communication in subcutaneous adipose tissue from patients with lymphedema inferred key signals involved in PACs. **(A)** Bar plots show the ranking of outgoing (left) and incoming (right) signals of PACs in adipose tissue from patients with lymphedema compared to healthy individuals. The rank of signals was based on differences in overall information flow, calculated by the total weights in the cellular network of each group. **(B)** Circle plots showing the inferred upregulated signaling network in lymphedema adipose tissue. The arrows and edge color represent the direction (source: target). The thickness of the edge indicates the sum of the weight of the signals between populations.

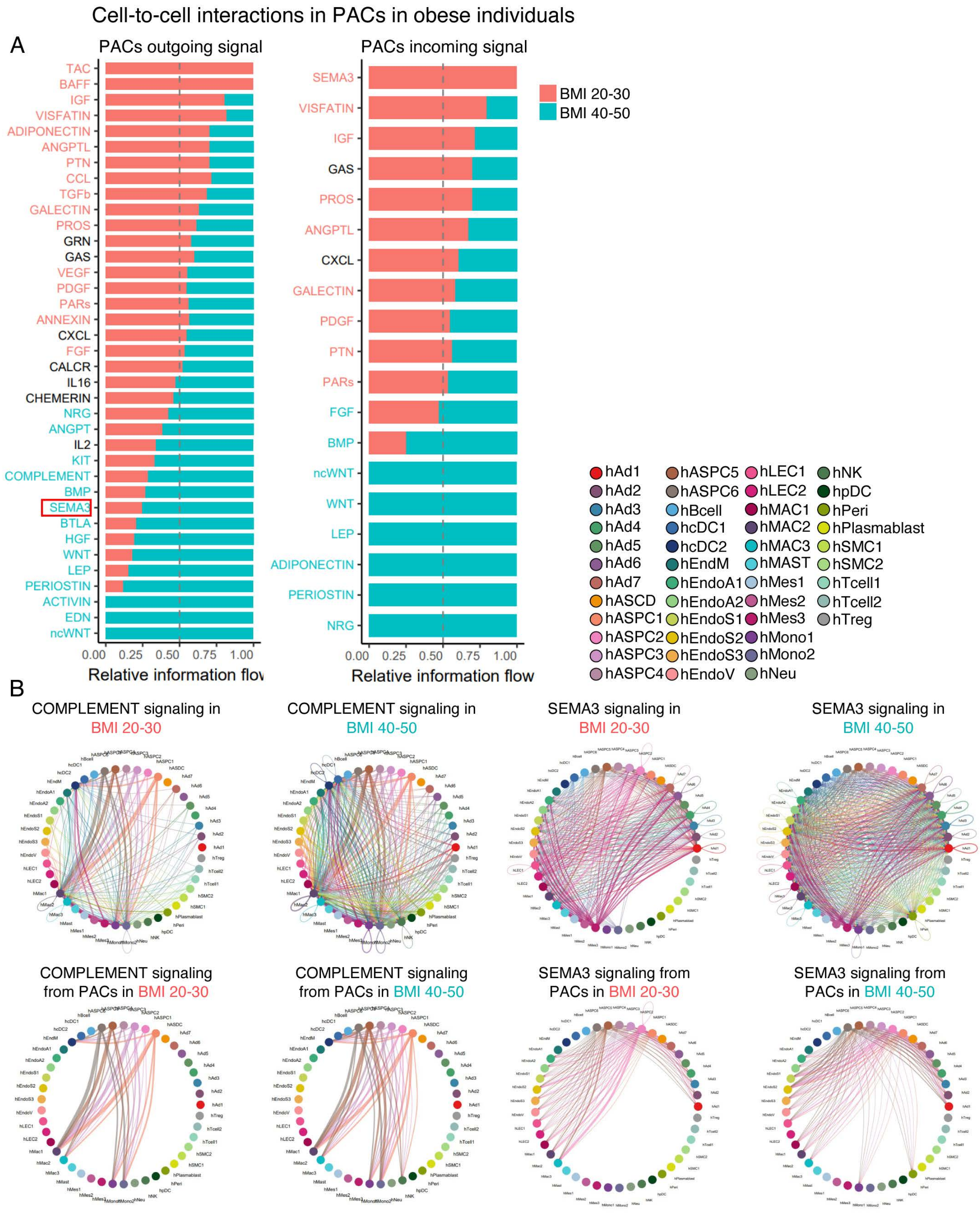

Supplementary Figure 8. Analysis of cell-cell communication in adipose tissue from obese individuals inferred key signals via PACs. **(A)** Bar plots show the ranking of outgoing (left) and incoming (right) signals of PACs in visceral adipose tissue (VAT) from obese individuals compared to overweight individuals. The rank of signals was based on differences in overall information flow, calculated by the total weights in the cellular network of each group. **(B)** Circle plots showing the inferred upregulated signaling network in obese VAT. The arrows and edge color represent the direction (source: target). The thickness of the edge indicates the sum of the weight of the signals between populations.

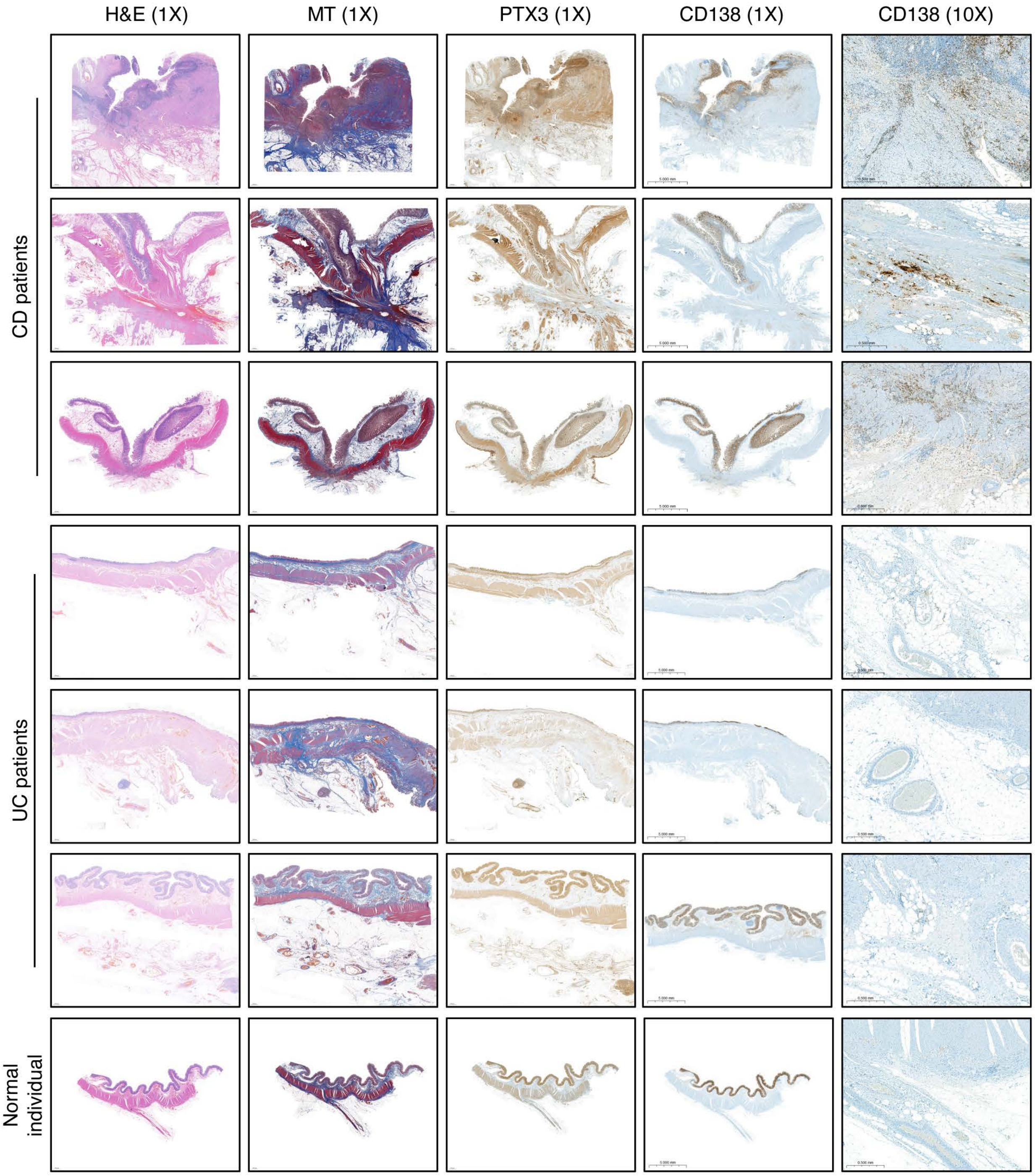

Supplementary Figure 9. Pentraxin-3 and CD138 expression is elevated in CrF. Images show histopathological evaluations of CrF in CD patients (n=3), iMAT in UC patients (n=3), and MAT from a normal individual (n=1). Hematoxylin-eosin (H&E), Masson trichrome (MT), Pentraxin-3 (PTX3) and Syndecan-1(CD138) staining, were presented separately.

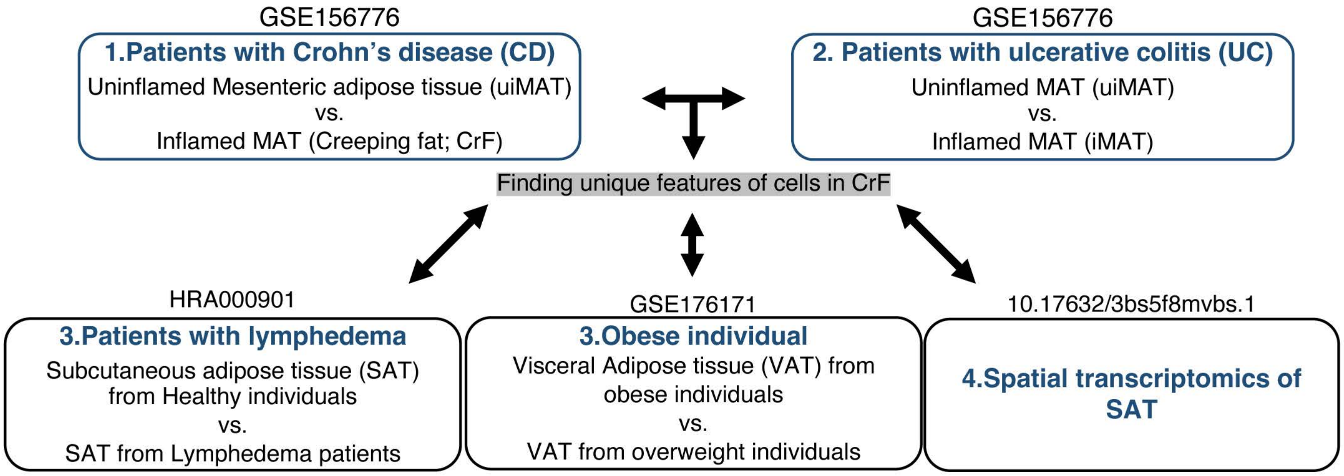

Supplementary Figure 10. A schema illustrating the datasets used for analysis and the comparative approach in this study
